# Supplementary material for: Neuronal HSF-1 coordinates the propagation of fat desaturation across tissues to enable adaptation to high temperatures in C. elegans
Source: PLoS Biol. 2021 Nov 1;19(11):e3001431. doi: 10.1371/journal.pbio.3001431 (PMC8585009; doi:10.1371/journal.pbio.3001431)
Supplement: S1 Fig — HSF-1, heat shock factor 1. (DOCX) [file pbio.3001431.s001.docx]

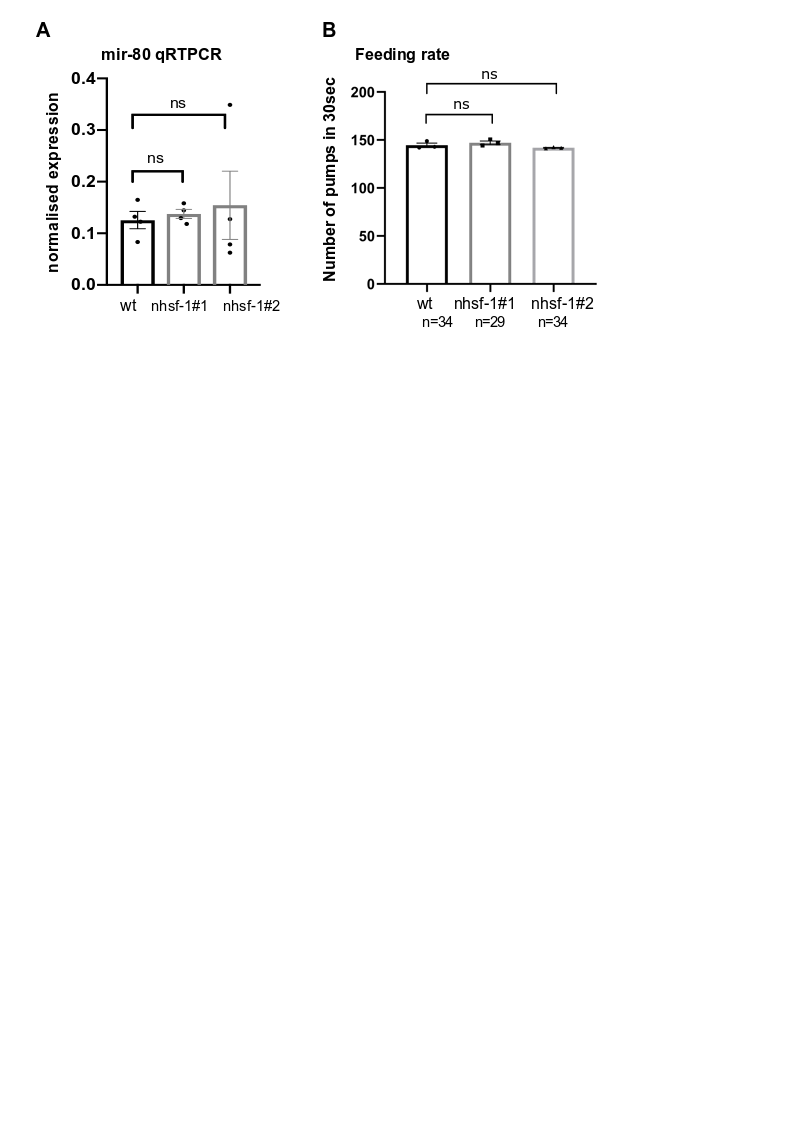


**Fig S1. Overexpression of *hsf-1* in neurons does not cause animals to experience starvation.**

(A-B) Ectopic expression of *hsf-1* in neurons does not cause starvation. (A) qRT-PCR shows that a known starvation marker, *mir-80,* is upregulated neither in *hsf-1*^neuro^*#*1 nor *hsf-1* ^neuro^ *#2* animals. Statistics were performed using a one-way ANOVA **(Table S8).** (B) Animals are feeding normally as they have pharyngeal pumping levels that are comparable to wild-type controls. All graphs show paired biological replicates where the bar represents SEM and n values is the number of worms. Young adult WT (N2) compared to *hsf-1*^neuro^*#*1 (MOC141) or *hsf-1* ^neuro^ *#2* (AGD1289). Statistics were performed using a one-way ANOVA. P-value WT vs *nhsf-1^neuro^ (line#1):*0.5637, P-value WT vs *nhsf-1^neuro^ (line#2):*0.5025. All data can be found in **Data_Figure_S1.**
