# Supplementary material for: Neuronal HSF-1 coordinates the propagation of fat desaturation across tissues to enable adaptation to high temperatures in C. elegans
Source: PLoS Biol. 2021 Nov 1;19(11):e3001431. doi: 10.1371/journal.pbio.3001431 (PMC8585009; doi:10.1371/journal.pbio.3001431)
Supplement: S2 Fig — HSF-1, heat shock factor 1; hsf-1neuro, neuronal overexpression of hsf-1; Lof, loss of function. (DOCX) [file pbio.3001431.s002.docx]

**
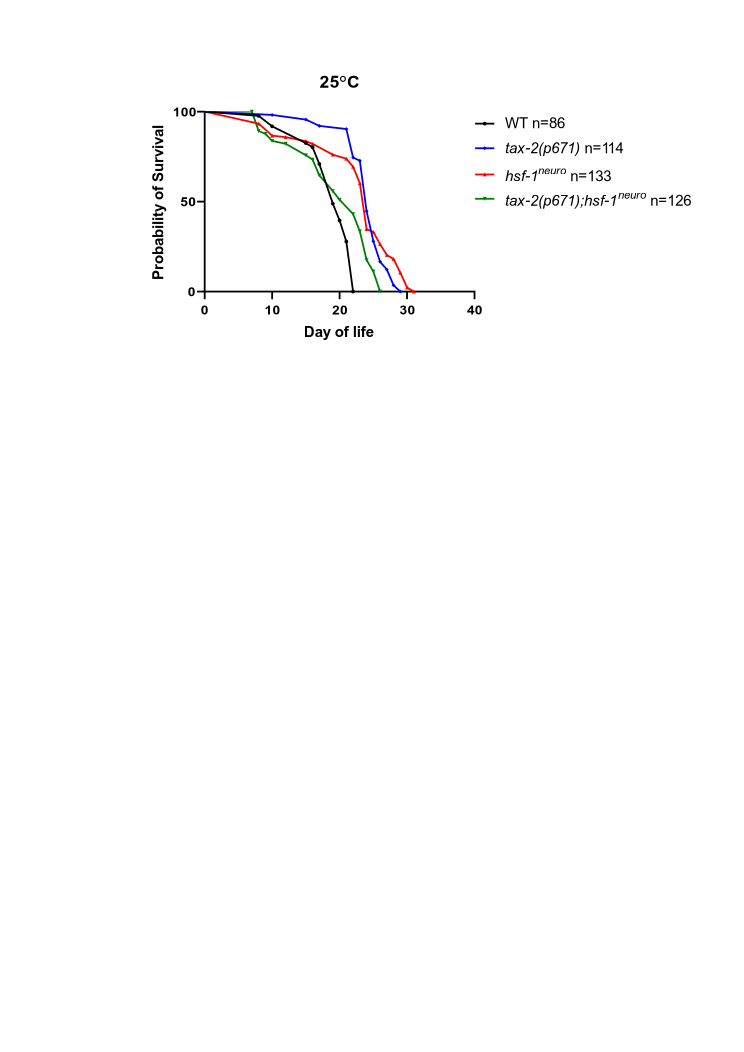
**

**Fig S2. Loss of function mutation in *tax-2* suppresses lifespan extension of *hsf-1^neuro^* at 25°C**. Representative survival curve of WT, *hsf-1^neuro^* line 2 (AGD1289), *tax-2(p671)* (MOC312) and *hsf-1^neuro^; tax-2(p671)* (MOC252) animals raised at 25°C. WT: 86 deaths, Median survival: 19 days. *hsf-1^neuro^*: 126 deaths, 8 censored, Median survival: 22 days. *tax-2(p671):* 114 deaths, median survival: 24 days. *tax-2(p671);hsf-1^neuro^*: 133 deaths, 2 censored, median survival: 24 days. P-value (log rank-test) WT vs *hsf-1^neuro^*: <0.0001, P-value WT vs *tax-2(p671)* <0.001, P-value WT vs *hsf-1^neuro^;tax-2(p671)* <0.0001. P-value *tax-2(p671)* vs *hsf-1^neuro^*= 0.2346, P-value *hsf-1^neuro^* vs *hsf-1^neuro^;tax-2(p671)* <0.0001, P-value *tax-2(p671)* vs *hsf-1^neuro^;tax-2(p671)* <0.0001. All data for biological replicates is in **Table S12** and **Data_Figure_S2.**
