# Supplementary material for: Neuronal HSF-1 coordinates the propagation of fat desaturation across tissues to enable adaptation to high temperatures in C. elegans
Source: PLoS Biol. 2021 Nov 1;19(11):e3001431. doi: 10.1371/journal.pbio.3001431 (PMC8585009; doi:10.1371/journal.pbio.3001431)
Supplement: S3 Fig — HSF-1, heat shock factor 1. (DOCX) [file pbio.3001431.s003.docx]

**
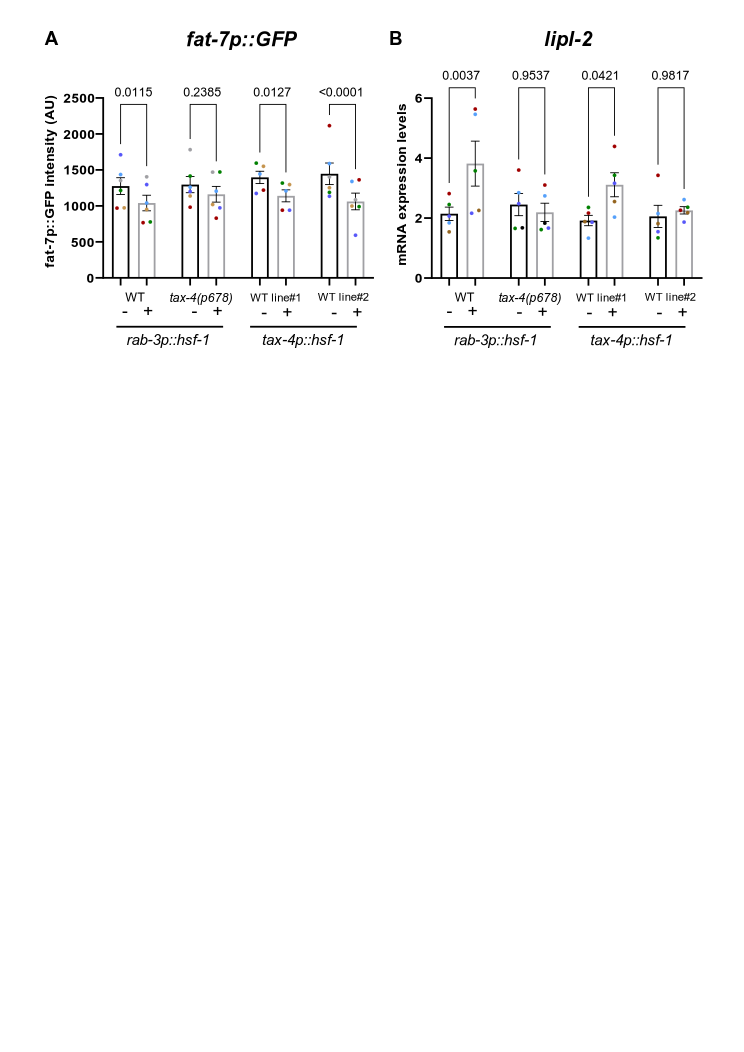
**

**Fig S3. Overexpression of *hsf-1* in *tax-4* expressing neurons is able to rescue *fat-7p::GFP* fluorescence but not lipases levels.** (A) Quantification of *fat-7p::GFP* fluorescence in WT (MOC193) and *tax-4(p678)* (MOC232) animals overexpressing extra-chromosomal *hsf-1* under the pan-neuronal *rab-3* promoter and in two extra-chromosomal lines overexpressing *hsf-1* driven by *tax-4* promoter; line #1 (MOC354), and line #2 (MOC353). The two lines overexpressing *hsf-1* in *tax-4* neurons exhibit significant decrease in *fat-7p::GFP* fluorescence in the presence of *tax-4p::hsf-1* overexpression and phenocopy animals overexpressing *hsf-1* in all neurons. Animals were monitored at day 3 of adulthood. P-values from pairwise multiple comparisons from Two-Way ANOVA (**Table S6**). Error bars: SEM, each dot represents a distinct biological replicate. Each biological replicate is color-coded. (B) Levels of lipase *lipl-2* mRNA expression measured by qRT-PCR in WT (MOC193) and *tax-4(p678)* (MOC232) animals overexpressing extra-chromosomal *hsf-1* under the pan-neuronal *rab-3* promoter and in two extra-chromosomal lines overexpressing *hsf-1* driven by *tax-4* promoter; line #1 (MOC354), and line #2 (MOC353). As described earlier, *lipl-2* mRNA expression increases significantly in WT worms overexpressing *hsf-1* in all neurons but not in *tax-4(p678)* background. In line #1 of animals overexpressing *hsf-1* in tax-4 neurons only, *lipl-2* levels are increased barely above significance in the presence of the transgene, whereas the presence of the *tax-4p::hsf-1* transgene does not affect *lipl-2* mRNA expression in line#2. We had similar results with *lipl-1* and *lipl-3* mRNA expression where levels remain unaffected by the *tax-4p::hsf-1* transgene. Animals were monitored at day 3 of adulthood. P-values from pairwise multiple comparisons from Two-Way ANOVA (**Table S8**). Error bars: SEM, each dot represents a distinct biological replicate. Each biological replicate is color-coded. All data can be found in **Data_Figure_S3.**
