# Supplementary material for: Neuronal HSF-1 coordinates the propagation of fat desaturation across tissues to enable adaptation to high temperatures in C. elegans
Source: PLoS Biol. 2021 Nov 1;19(11):e3001431. doi: 10.1371/journal.pbio.3001431 (PMC8585009; doi:10.1371/journal.pbio.3001431)
Supplement: S4 Fig — BMP, bone morphogenetic protein; HSF-1, heat shock factor 1; TGF-β, transforming growth factor ß. (DOCX) [file pbio.3001431.s004.docx]

**
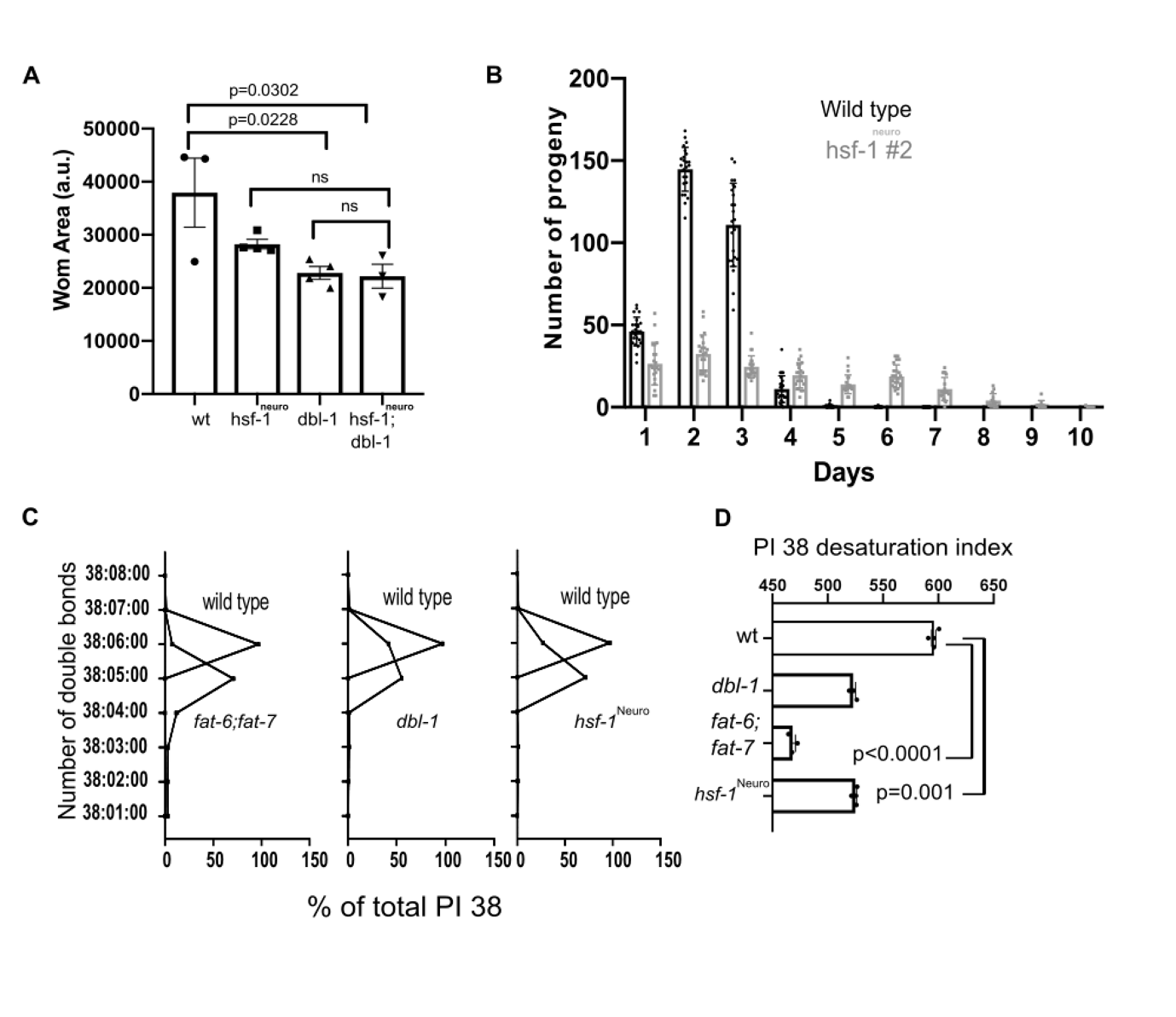
**

**Fig S4. Overexpression of *hsf-1* in neurons phenocopies TGF-β/BMP mutants.**

(A) The small size of *hsf-1^neuro^#2* is caused by reduced TGF-β/BMP signalling. Area in arbitrary units (a.u.) of the genotypes: wildtype (N2); *hsf-1^neuro^#2* (AGD1289); *dbl-1(nk-3)* (NU3); *hsf-1^neuro^#2*; *dbl-1(nk3)*(MOC253). BMP is epistatic to *nhsf-1* because the double mutants are not shorter than any single mutant alone. Each dot corresponds to a distinct biological replicate, error bar corresponds to the SEM, and statistics were performed using a one-way ANOVA test. (B) *hsf-1^neuro^#2* causes a germline senescence phenotype. A representative biological replicate showing the number of self-progeny laid per day at 20℃ from day 1 to 10 of adulthood in *hsf-1^neuro^#2* (AGD1289, grey bars) and wildtypes (N2, black bars). Each dot represents the total progeny output of a single worm.  **Table S9** shows that the difference in reproductive output is significantly lower than WT during the first 3 days of adulthood and then remains higher than wt for the last two tested days (two-way ANOVA interaction, P-value= 0.0293. (C-D) Changes in the lipid composition of *dbl-1* animals are consistent with decreased saturation and fluidity in the plasma membrane. (C) Relationship between the quantity (in ng/ng DNA) of acyl chain (carbon number related to the FA composition in position *sn-1* and *sn-2* in glycerophosphoplids) for the specified number of double bonds (from 0, saturated to 8, poly-unsaturated) in PI 38 (phosphatidylinositol with an acyl chain of 38 carbons) All normalised data is listed in **Table S1.** (D) Shows desaturation index. Steroyl-CoA desaturase mutants: *fat-6/7*=*fat-6 (tm33)1; fat-7 (wa36); dbl-1= dbl-1(nk3)*(NU3)*; hsf-1^neuro^*(AGD1289)*.* All normalised data is listed in **Table S2** and all data in in **Data_Figure_S4.**
