# Supplementary material for: Neuronal HSF-1 coordinates the propagation of fat desaturation across tissues to enable adaptation to high temperatures in C. elegans
Source: PLoS Biol. 2021 Nov 1;19(11):e3001431. doi: 10.1371/journal.pbio.3001431 (PMC8585009; doi:10.1371/journal.pbio.3001431)
Supplement: S5 Fig — BMP, bone morphogenetic protein; HSF-1, heat shock factor 1; hsf-1neuro, neuronal overexpression of hsf-1; TGF-β, transforming growth factor ß. (DOCX) [file pbio.3001431.s005.docx]

**
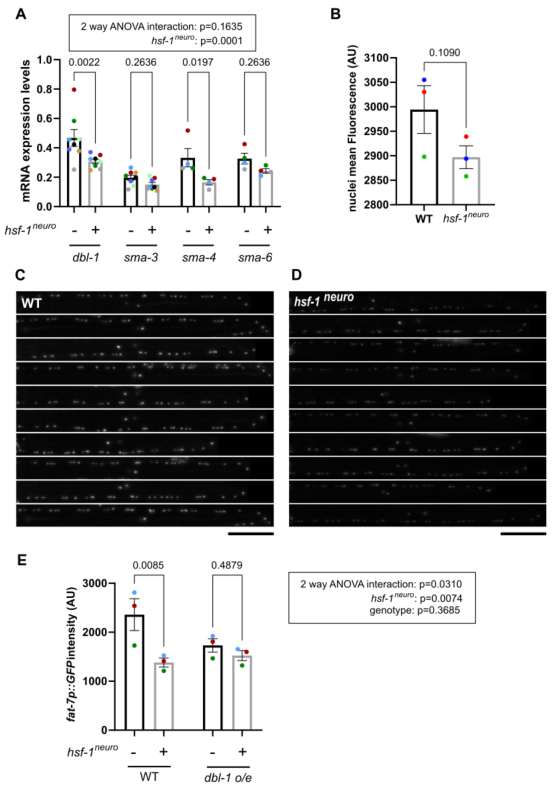
**

**Fig S5. Neuronal overexpression of *hsf-1* decreases the activity of TGF-β/BMP signalling.** (**A**) Levels of *dbl-1*, *sma-3*, *sma-4* and *sma-6* mRNA expression measured by qRT-PCR in WT (N2) and *hsf-1*^neuro^(AGD1289). *Sma-3(R-SMAD)* and *sma-4 (Co-SMAD)* encode intracellular signaling transducers of TGF-β/BMP signaling pathway, while *sma-6* encodes a Type-I receptor of TGF-β/BMP signaling (1). The effect of neuronal stress is similar for all the genes tested: p-value interaction *hsf-1^neuro^* and gene tested=0.1635 (ns), two-way ANOVA, **Table S8**. Across all four target genes, neuronal stress significantly decreases their mRNA levels: p-value for factor *hsf-1^neuro^*=0.0001 (***), Two-way ANOVA, **Table S8**. P-values from pairwise multiple comparisons from Two-Way ANOVA (**Table S8**). Animals were harvested at young adult stage. Error bars: SEM, each dot represents a distinct biological replicate. Each biological replicate is color-coded. (**B-D**) In the presence of *hsf-1^neuro^*, the GFP fluorescence of a *dbl-1* transcriptional reporter is reduced, although not significantly. (**B**) Quantification of the fluorescence from the *ctIs43[dbl-1p::GFP+dbl-1p::GFP::NLS]* transcriptional reporter (2) in WT(BW1935) and *hsf-1^neuro^*(MOC372) background. Animals were harvested at young adult stage. Error bars: SEM, each dot represents a distinct biological replicate. Each biological replicate is color-coded. P-values were obtained from a paired t-test (**Table S6**). (**C-D**) Images of the *ctIs43[dbl-1p::GFP+dbl-1p::GFP::NLS]* transcriptional reporter, expressed in the ventral nerve cord of WT(BW1935) and *hsf-1^neuro^*(MOC372). Scale bar=100µm. (**E**) Overexpression of *dbl-1* does not rescue *fat-7p::GFP* levels. Quantification of *fat-7p::GFP* fluorescence in WT (MOC193), and in animals carrying the *dbl-1p::dbl-1::TM::mcherry* transgene which causes worms to be longer (3) (MOC377), +/- *hsf-1^neuro^*). There is an interaction between neuronal stress and genotypes (2 way ANOVA interaction: p-value=0.0310), meaning that effect of neuronal stress affects differently the two genotypes. Overall, regardless of genotypes, *hsf-1^neuro^* decreases fat-7p::GFP (p-value *hsf-1^neuro^* factor=0.0074). However, adjusted pairwise comparison of *dbl-1 o/e* animals shows no reduction of *fat-7p::GFP* fluorescence in the presence of *hsf-1^neuro^* (p-value=0.4879, as compared to p=0.0085 in WT animals). Regardless of neuronal stress, there is no difference between WT and *dbl-1o/e* (p-value genotype factor=0.3685). Animals were imaged at day 3 of adulthood. P-values from pairwise multiple comparisons from Two-Way ANOVA (**Table S6).** Error bars: SEM, each dot represents a distinct biological replicate. Each biological replicate is color-coded. All data can be found in **Data_Figure_S5.**

**References**

1. Savage-Dunn C. TGF-β signaling. WormBook. 2005;1–12.

2. Suzuki Y, Yandell MD, Roy PJ, Krishna S, Savage-Dunn C, Ross RM, et al. A BMP homolog acts as a dose-dependent regulator of body size and male tail patterning in *Caenorhabditis elegans*. Development. 1999;126(2):241–50.

3. Zhang X, Zhang Y. DBL-1, a TGF-β, is essential for *Caenorhabditis elegans* aversive olfactory learning. Proc Natl Acad Sci U S A. 2012;109(42):17081–6.
