# Supplementary material for: Neuronal HSF-1 coordinates the propagation of fat desaturation across tissues to enable adaptation to high temperatures in C. elegans
Source: PLoS Biol. 2021 Nov 1;19(11):e3001431. doi: 10.1371/journal.pbio.3001431 (PMC8585009; doi:10.1371/journal.pbio.3001431)
Supplement: S6 Fig — (A) Neurons expressing tax-4p::tax-4::wrmScarlet are adjacent to neurons where HSF-1 is activated. (B) Neurons expressing tax-4p::his-24::mOrange are adjacent to neurons where HSF-1 is activated, with occasional overlap. (C) Epistasis analysis suggest that tax-2 plays roles beyond the modulation of TGF-β signaling. HSF-1, heat shock factor 1; TGF-β, transforming growth factor ß. (DOCX) [file pbio.3001431.s006.docx]

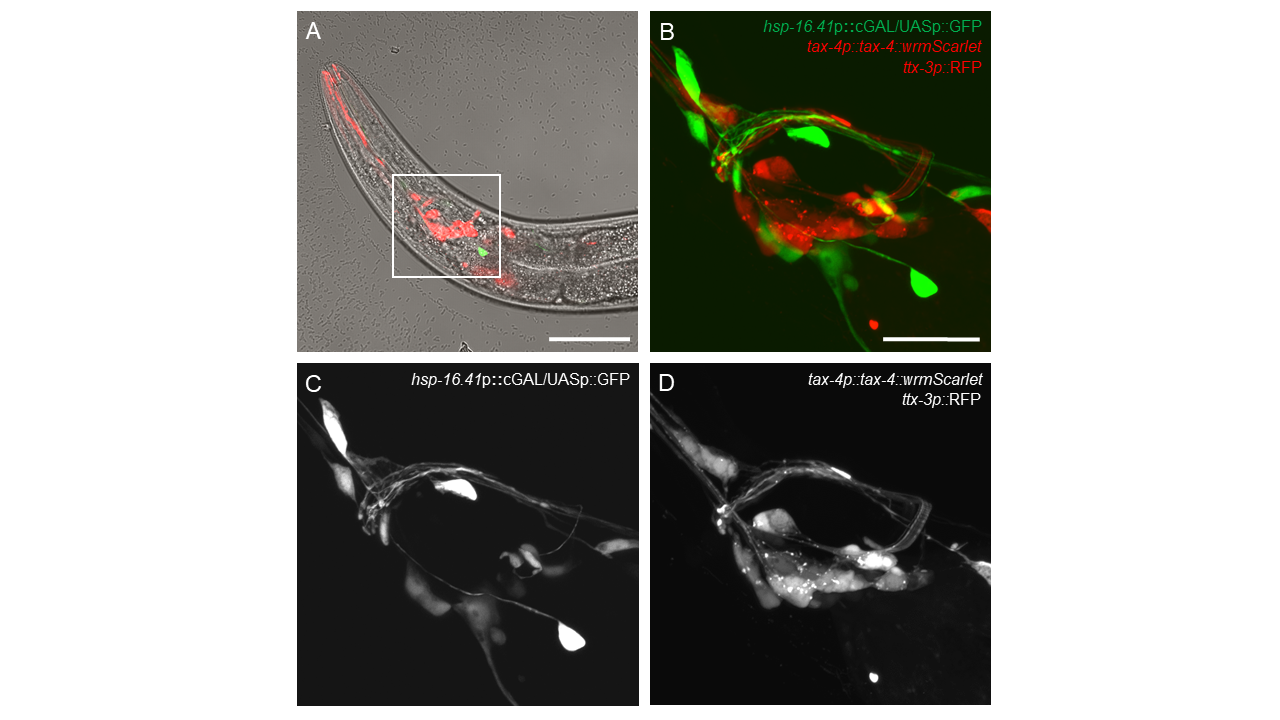


**Fig S6A. Neurons expressing *tax-4p::tax-4::wrmScarlet*  are adjacent to neurons where HSF-1 is activated.** (A-D) Micrographs of animals carrying both *tax-4p::tax-4::wrmScarlet* (1) and *hsp-16.41p::cGAL/UASp::GFP* transgenes with the co-injection marker *ttx-3p*::RFP, expressed in the AIY neuron (2). Green neurons expressing *hsp-16.41p::cGAL/UASp::GFP* depict neurons where HSF-1 is activated (neuronal stress). While no direct co-localization was observed in this series of experiments between neurons expressing *tax-4* and neurons where HSF-1 is activated, these images reveal that the two neuronal circuits are intricate and in close proximity. Animals were grown at 25°C and imaged at day 2 of adulthood. Images were taken on a Zeiss confocal microscope at 60X objective, left is anterior. (A) DIC, scale bar=50 µm. (B) Composite image of maximum projection of the zone delimited by a square in A. scale bar = 20 µm. (C) and (D) show respectively the green and red channels of the maximum projection in (B).

**
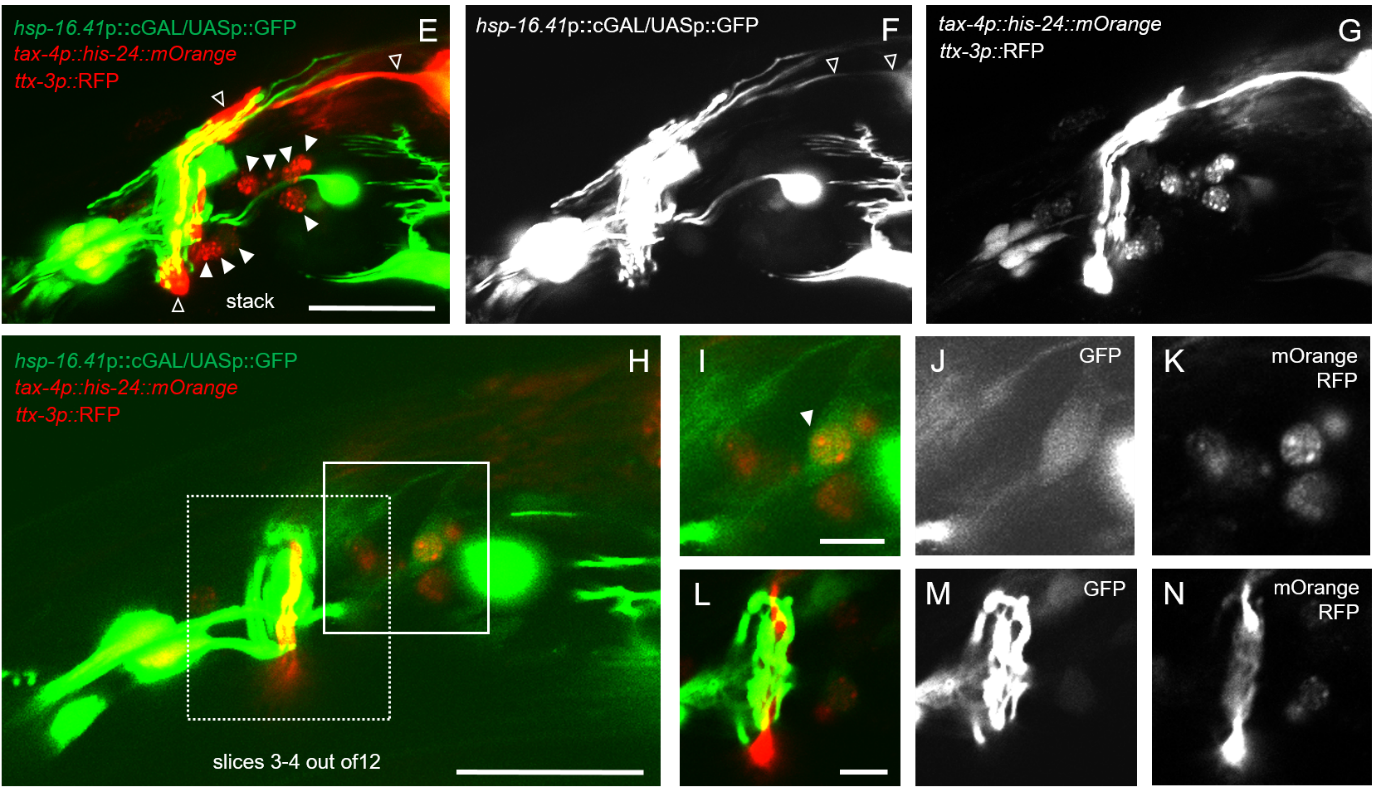
**

**Fig S6B. Neurons expressing *tax-4p::his-24::mOrange*  are adjacent to neurons where HSF-1 is activated, with occasional overlap.** (E-N) Micrographs of animals carrying both *tax-4p::his-24::mOrange* and *hsp-16.41p::cGAL/UASp::GFP* transgenes with the co-injection marker *ttx-3p*::RFP, expressed in the AIY neuron (2). This series of experiments showed again that neurons expressing *tax-4* and neurons where HSF-1 is activated are in close proximity and occasionally overlap, as seen in I. Green neurons expressing *hsp-16.41p::cGAL/UASp::GFP* depict neurons where HSF-1 is activated (neuronal stress). Red nuclei (full arrowhead in E) depict *tax-4* expressing neurons, while red neurons in E is AIY (*ttx-3p*::RFP co-injection marker), shown by empty arrowhead in E. The expression levels of *tax-4p::his-24::mOrange* were very low in comparison to *ttx-3p:RFP*. In (E-G), we observed overlap of neurons where HSF-1 is activated with AIY (empty arrowhead in F). (E) Composite image of maximum projection, Stack of 12 slices, (F) maximum projection, GFP channel, (G) maximum projection, red channel. (H-N) Neurons where HSF-1 is activated occasionally overlap with TAX-4 expressing neurons, as seen in (I). (H-N) Maximum projection of slices 3 and 4 out of the stack of 12 from (E-G), where GFP intensity has been increased reveals overlap between . *hsp-16.41p::cGAL/UASp::GFP* and *tax-4p::his-24::mOrange* in one neuron (full arrowhead in I). (I-K) Magnification of zone delimited by full line in H. (I) composite image, (J) green channel), (K) red channel. (L-N) Maximum projection of slices 3 and 4 out of the stack of 12 from (E-G) also shows that in this animal green neurons where HSF-1 is activated are wrapped around the AIY neuron in red (*ttx-3p*::RFP marker) but do not directly overlap. (L-N) Magnification of zone delimited by dashed line in H. (L) composite image, (M) green channel), (N) red channel. Animals were grown at 25°C and imaged at day 2 of adulthood. Images were taken on a Zeiss confocal microscope at 60X objective, which enabled to perform differential spectral analysis of both mOrange and RFP fluorophores. left is anterior. (E, H) scale bar=20 µm. (I, L) scale bar=5 µm.

­­


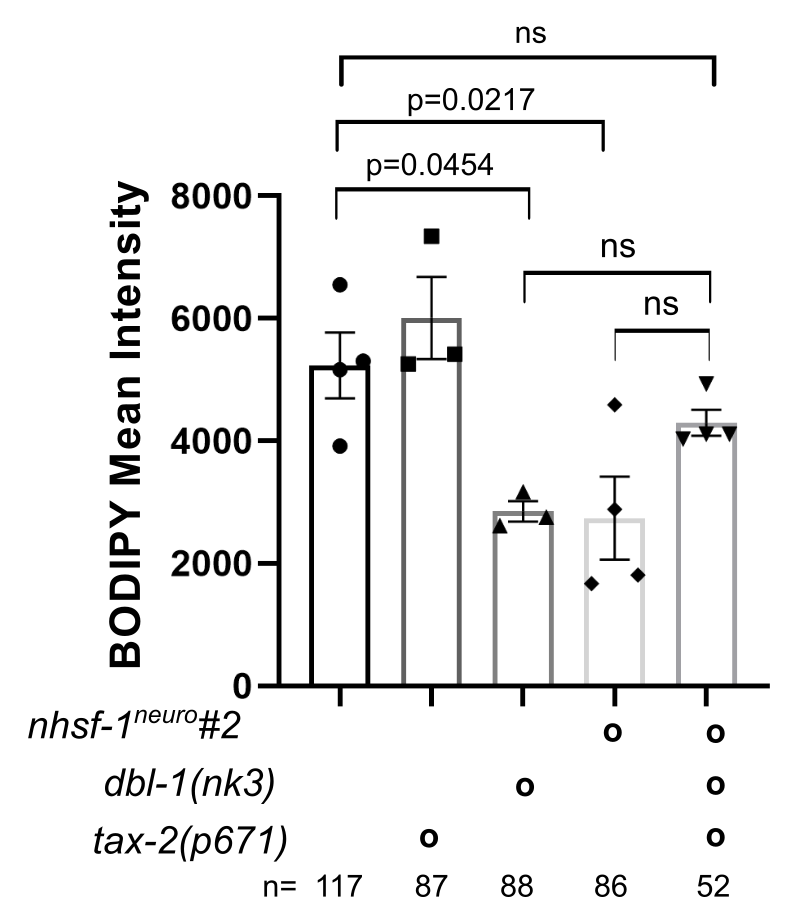


**Fig S6C. Epistasis analysis suggest that *tax-2* plays roles beyond the modulation of TGF-beta signaling.**

Lipid droplets (LD) were quantified using BODIPY staining in: wild type; *tax-2(p671)* (PR671), *dbl-1(nk-3)* (NU3);  *hsf-1^neuro^* *#2* (AGD1289); and *hsf-1^neuro^#2; tax-2(p671); dbl-1(nk3)* (MOC253). Data and statistic can be found in **Table S5**. Each dot corresponds to a biological replicate, the bar indicates the SEM and n correspond to total number of worms. If the suppression of the lean phenotype of  *hsf-1^neuro^*  caused by mutations in t*ax-2/4* was solely dependent on the reversion of TGF-β/BMP signalling to wild type levels, then *tax-2/4* mutations should be unable to rescue  *hsf-1^neuro^* lean phenotype in the total absence of *dbl-1* function. We find, however, that a *tax-2* mutation is still able to rescue LD accumulation of  *hsf-1^neuro^* animals that lack *dbl-1* function. Because the triple mutant has lower LD accumulation than wild type animals, we suggest that TAX-2/4 neurons rely on other mechanisms in addition to TGF-β/BMP signalling to alter fat metabolism. This idea is consistent with the observation that *dbl-1* causes a decrease in *fat-7/6* but does not alter lipase levels (**Table S8**), indicating two independent branches of gene regulation.

All data can be found in **Data_Figure_S6.**

**References**

1. Fasseas MK, Grover M, Drury F, Essmann CL, Kaulich E, Schafer WR, et al. Chemosensory Neurons Modulate the Response to Oomycete Recognition in Caenorhabditis elegans. Cell Rep [Internet]. 2021;34(2):108604. Available from: https://doi.org/10.1016/j.celrep.2020.108604

2. Bertrand V, Hobert O. Linking Asymmetric Cell Division to the Terminal Differentiation Program of Postmitotic Neurons in C. elegans. Dev Cell. 2009;16(4):563–75.
