# Supplementary material for: Neuronal HSF-1 coordinates the propagation of fat desaturation across tissues to enable adaptation to high temperatures in C. elegans
Source: PLoS Biol. 2021 Nov 1;19(11):e3001431. doi: 10.1371/journal.pbio.3001431 (PMC8585009; doi:10.1371/journal.pbio.3001431)
Supplement: S7 Fig — BMP, bone morphogenetic protein; TGF-β, transforming growth factor ß. (DOCX) [file pbio.3001431.s007.docx]

**
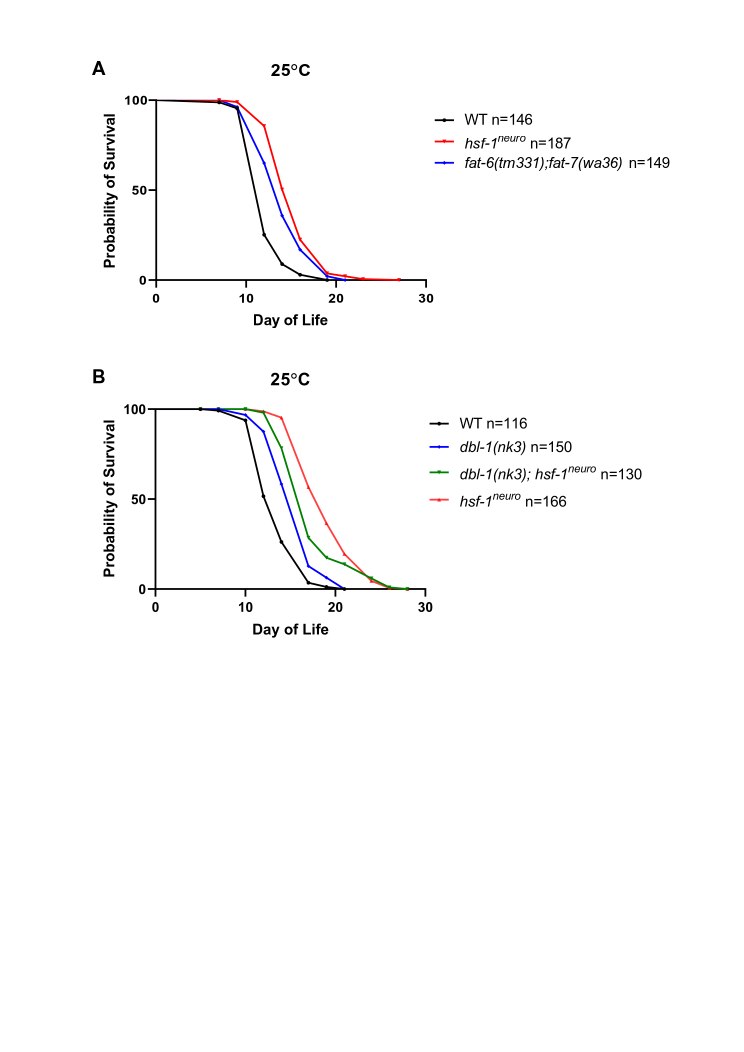
**

**Fig S7. The activity of TGF-β/BMP and fat desaturases is detrimental to survival at 25°C.** (**A**) Representative survival curve of WT, *hsf-1^neuro^* line 2 (AGD1289), *and fat-6(tm331);fat-7(wa36)* (BX156) animals raised at 25°C. The curves show that removing desaturases *fat-6/fat-7* function, extends lifespan at 25°C, phenocopying hsf-1^neuro^. WT: 146 deaths, 14 censored, median survival: 12 days. *hsf-1^neuro^*: 187 deaths, 14 censored, median survival: 16 days, *fat-6(tm331);fat-7(wa36):* 149 deaths, 30 censored, median survival: 14 days. P-value (log rank-test) WT vs *hsf-1^neuro^*: <0.0001, p-value WT vs *fat-6(tm331);fat-7(wa36)* <0.0001, p-value *hsf-1^neuro^* vs *fat-6(tm331);fat-7(wa36)=0.0012*. (**B**) Representative survival curve of WT, *hsf-1^neuro^* line 2 (AGD1289), *dbl-1(nk3)* (NU3) and *hsf-1^neuro^;dbl-1(nk3)* (MOC254) animals raised at 25°C. The curves show that removal of *dbl-1(nk3)* is beneficial for survival at 25°C, but not as beneficial as *hsf-1^neuro^.* This result is to be expected as *dbl-1(nk3)* makes worms more susceptible to bacterial pathogenesis (1). WT: 146 deaths, 24 censored, median survival: 14 days. *hsf-1^neuro^*: 166 deaths, 33 censored, median survival: 19 days, *dbl-1(nk3):* 150 deaths, 50 censored, median survival:17, *hsf-1^neuro^;dbl-1(nk3)*: 130 deaths, 34 censored, median survival:17 days. P-value (log rank-test) WT vs *hsf-1^neuro^* <0.0001, p-value (log rank-test) WT vs *dbl-1(nk3)* <0.0001, p-value WT vs *hsf-1^neuro^;dbl-1(nk3)* <0.0001, p-value *dbl-1(nk3)* vs *hsf-1^neuro^* <0.0001, p-value *hsf-1^neuro^*;*dbl-1(nk3)* vs *hsf-1^neuro^* <0.0001, p-value *dbl-1(nk3)* vs *hsf-1^neuro^;dbl-1(nk3)* <0.0001. Lifespan assays were performed on FUDR, as *dbl-1(nk3)* mutants are susceptible to the pathogenic effects of OP50 (1). All data for biological replicates is in **Table** **S12** and all data can be found in **Data_Figure_S12.**

**References**

1. Gumienny TL, Savage-Dunn C. TGF-β signaling in C. elegans. WormBook. 2013;1–34.
