# Supplementary material for: Neuronal HSF-1 coordinates the propagation of fat desaturation across tissues to enable adaptation to high temperatures in C. elegans
Source: PLoS Biol. 2021 Nov 1;19(11):e3001431. doi: 10.1371/journal.pbio.3001431 (PMC8585009; doi:10.1371/journal.pbio.3001431)
Supplement: S8 Fig — HSF-1, heat shock factor 1. (DOCX) [file pbio.3001431.s008.docx]

**
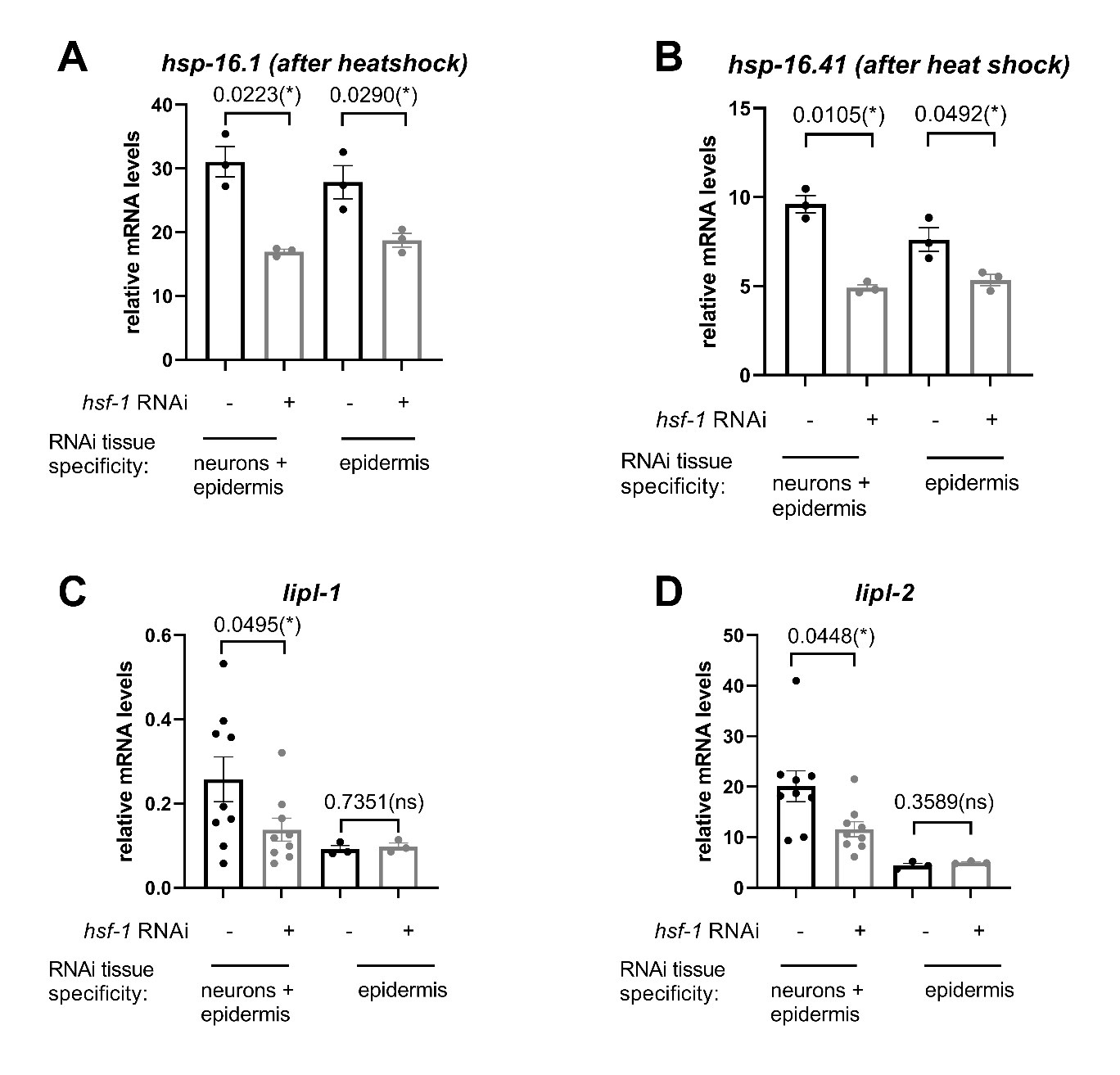
**

**Figure S8. Neuronal and not epidermis expression of *hsf-1* is necessary for lipase expression at 25°C.** Relative expression levels of *hsp16.1*(A), *hsp16.41*(B), *lipl-1*(C) and *lipl-2*(D) mRNA by qRT-PCR Two strains were used: TU3401*(sid-1(pk3321)V,uIs69 [pCFJ190(myo-2p::mCherry*) + *unc-119p*::*sid-1*) which is primarily a pan neuronal RNAi sensitive strain with some activity in the epidermis and QK52: *rde-1(n219); xkls99[wrt-2p::red-1::unc-543’UTR]* which is an epidermis-specific RNAi sensitive strain. Animals were raised at 25°C for 1 generation and were fed either L4440 (empty vector, EV) control or *hsf-1*double stranded RNA (Ahringer RNAi library) for 48h, from late L4 onwards. To ascertain the effectiveness of the RNAi treatment we looked at the effect of *hsf-1* KD on the heat shock response. Animals were placed under heat shock conditions and the expression of chaperones (*hsp16.1* and *hsp16.41)* were tested 30 minutes after the heat shock (see materials and methods for details). As expected. KD of *hsf-1* in both neurons and epidermis causes a dampening in the heat shock response. Levels of mRNA levels were measured at day 3 of adulthood. Statistics were performed using paired t-test (**Table S8**). Bars represent the SEM and each dot represents a biological replicate. All data can be found in **Data_Figure_S8.**
