# Supplementary material for: Neuronal HSF-1 coordinates the propagation of fat desaturation across tissues to enable adaptation to high temperatures in C. elegans
Source: PLoS Biol. 2021 Nov 1;19(11):e3001431. doi: 10.1371/journal.pbio.3001431 (PMC8585009; doi:10.1371/journal.pbio.3001431)
Supplement: S9 Fig — HSF-1, heat shock factor 1. (DOCX) [file pbio.3001431.s009.docx]

**Summary Figure. An HSF-1 dependent thermostat to control organismal adaptation to warmer temperatures.** The constitutive activation of neuronal HSR, causes animals to perceive a higher temperature than the one they are actually experiencing and to mount a program that is normally used by wild type animals at 25°C. HSF-1 is necessary to control at least part of the temperature-dependent fat remodelling program and disruption hsf-1 function in neurons causes animals to survive less at 25°C. The model is that sensory neurons act as a thermostat, because they are exquisitely sensitive to ambient temperatures and activate and HSF-1 dependent stress response when animals are raised at 25°C, and not at 15°C. This slight increase in temperature is enough to disturb the fluidity of the plasma membrane and requires the activation of corrective homeoviscous adaptive mechanisms. These transcriptional changes cause intestinal cells to produce less unsaturated fats and to mobilize FA stores from lipid droplets. The net result is an increase in the number of phospholipids produced and a change in the saturation of the FAs, which corrects the fluidity of the plasma membranes across all cells in the body and ensures viability at warmer temperatures. Additional information in the mechanism is shown in **Figure 6**.
