# Supplementary material for: Neuronal HSF-1 coordinates the propagation of fat desaturation across tissues to enable adaptation to high temperatures in C. elegans
Source: PLoS Biol. 2021 Nov 1;19(11):e3001431. doi: 10.1371/journal.pbio.3001431 (PMC8585009; doi:10.1371/journal.pbio.3001431)
Supplement: S4 Table — DE, differentially expressed; HSF-1, heat shock factor 1; hsf-1neuro, neuronal overexpression of hsf-1. (DOCX) [file pbio.3001431.s013.docx]

**S4 Table - Probability of overlap calculated using a hypergeometric distribution between DE genes from AGD1289 (hsf-1^neuro^#2) *vs* N2/ N2 at 25°C vs N2 at 15°C.** l2fc: log 2fold change

|  | l2fc=0 | l2fc=0.5 | l2fc = 1 | l2fc = 1.5 | l2fc=2 |
| --- | --- | --- | --- | --- | --- |
| Total genes with expression >1 in at least 1 dataset (Seqmonk quantitation) | 11,785 | 11,785 | 11,785 | 11,785 | 11,785 |
| DE genes RNA-seq AGD1289 vs N2 | 2,136 | 1,506 | 592 | 304 | 168 |
| DE genes temperature RNA-seq  N2 at 25°C vs N2 at 15°C | 1,089 | 637 | 345 | 202 | 133 |
| Overlapping DE genes | 284 | 1.24E+02 | 3.70E+01 | 1.60E+01 | 7.00E+00 |
| Expected common DE | 197 | 81 | 17 | 5 | 2 |
| Observed / Expected | 1.44 | 1.52 | 2.13 | 3.07 | 3.69 |
| P-value | 3E-12 | 3.41E-07 | 4.22E-06 | 1.87E-05 | 0.000625 |
