# Supplementary material for: Neuronal HSF-1 coordinates the propagation of fat desaturation across tissues to enable adaptation to high temperatures in C. elegans
Source: PLoS Biol. 2021 Nov 1;19(11):e3001431. doi: 10.1371/journal.pbio.3001431 (PMC8585009; doi:10.1371/journal.pbio.3001431)
Supplement: S5 Table — (DOCX) [file pbio.3001431.s014.docx]

**S5 Table: BODIPY intensity measurements**

| **Related to figure** | **Strain** | **Average**  **(+/-SEM)** | **Nb of worms** | **BR** | **% changes vs CTR** | **p value (*vs* CTR)** | **p value**  **(*vs* condition)** | **Statistical test** |
| --- | --- | --- | --- | --- | --- | --- | --- | --- |
| 2E | N2/CTR | 4232 (79) | 180 | 7 |  |  |  | paired t-test |
| 2E | AGD1289 (*hsf-1^neuro^#2*) | 2554 (268) | 173 | 7 | 40% DOWN | 0.0003 (***) |  |  |
| 2G | N2/CTR | 4731 (340) | 111 | 3 |  |  |  | paired t-test |
| 2G | MOC141 (*hsf-1^neuro^#1*) | 3343 (480) | 115 | 3 | 30% DOWN | 0.0343(*) |  |  |
| 3F | N2/CTR | 4254 (137.6) | 200 | 10 |  |  |  | Ordinary one-way ANOVA |
| 3F | AGD1289 (*hsf-1^neuro^#2*) | 2133 (311.9) | 101 | 7 | 50% DOWN | <0.0001 (****) | <0.0001 (****) vs PR671 | with Tukey's test |
| 3F | PR671( *tax-2(p671))* | 4654 (161.3) | 117 | 6 | 9% UP | 0.7370 (ns) | 0.0042 (**) vs MOC293 |  |
| 3F | MOC252 ( *tax-2(p671);*  *hsf-1^neuro^ #2)* | 4509 (170.4) | 100 | 5 | 6% UP | 0.9594 (ns) | <0.0001 (****) vs AGD1289 |  |
| 3F | PR694: *tax-2(p694)* | 3636 (517.5) | 87 | 3 | 14% DOWN | 0.5593 (ns) |  |  |
| 3F | MOC293 (*tax-2(p694);*  *hsf-1^neuro^#2)* | 3037 (96.97) | 74 | 3 | 29% DOWN | 0.0289 (*) |  |  |
| 4D | N2/CTR | 4256 (222.4) | 131 | 6 |  |  |  | RM one-way ANOVA |
| 4D | AGD1289 (*hsf-1^neuro^#2*) | 2290 (319.0) | 80 | 6 | 46% DOWN | 0.0052 (**) |  | with Tukey's test |
| 4D | NU3 (*dbl-1(nk3))* | 3177 (383.8) | 114 | 6 | 25% DOWN | 0.2318 (ns) |  |  |
| 4D | MOC254 (*dbl-1(nk3),*  *hsf-1^neuro^ #2)* | 2520 (356.1) | 121 | 6 | 40 % DOWN | 0.0163 (*) |  |  |
| S6C | N2/CTR | 5232 (538.6) | 117 | 4 |  |  |  | Ordinary one-way ANOVA |
| S6C | AGD1289 (*hsf-1^neuro^#2*) | 2740 (674.1) | 86 | 3 | 48% DOWN | 0.0217 (*) | vs PR671 p=0.0055 (**) | with Tukey's test |
| S6C | NU3 (*dbl-1(nk3))* | 2848 (167.2) | 88 | 3 | 46% DOWN | 0.0454 (*) | vs PR671 p=0.0115 (*) |  |
| S6C | PR671 (*tax-2(p671))* | 6007 (671.4) | 87 | 3 | 15% UP | 0.8324 (ns) |  |  |
| S6C | MOC253 (*tax-2(p671); dbl-1(nk3); hsf-1^neuro^#2)* | 4296 (212.9) | 52 | 4 | 18% DOWN | 0.6621 (ns) | vs AGD1289 p= 0.2192 (ns) |  |

Nb: number; CTR: control; N2 is the wild type strain; SEM: standard error of the mean
