# Supplementary material for: Neuronal HSF-1 coordinates the propagation of fat desaturation across tissues to enable adaptation to high temperatures in C. elegans
Source: PLoS Biol. 2021 Nov 1;19(11):e3001431. doi: 10.1371/journal.pbio.3001431 (PMC8585009; doi:10.1371/journal.pbio.3001431)
Supplement: S9 Table — HSF-1, heat shock factor 1; hsf-1neuro, neuronal overexpression of hsf-1. (DOCX) [file pbio.3001431.s018.docx]

| **Genotype** | **Assay** | **neuron(s) tested** | **Comparison *+* vs *- nhsf-1*** | **Significant?**  One-way ANOVA unless otherwise stated | | **Neuron(s) involved?** |
| --- | --- | --- | --- | --- | --- | --- |
| WT *+/- hsf-1^neuro^* | Bodily levels Fig 3F3-K | negative control | 50 % decrease | <0.0001 | | _ |
| *tax-2(p671)lof* *+/- hsf-1^neuro^* |  | positive control | 3% decrease | 0.9994 | | _ |
| *tax-2(p694) +/- hsf-1^neuro^* |  | tax-2(+) in **AWC**+**ASI**+**ASJ**  +**ASG**+**AWB**+**ASK** | 25% decrease | 0.2769  *tax-2(p694)+hsf-1^neuro^* vs  *hsf-1^neuro^*: 0.1629  *tax-2(p694)+hsf-1^neuro^* vs *tax-2(p671)+hsf-1^neuro^*: 0.1203 | | yes partially |
| **Genotype** | **Assay** | **neuron(s) tested** | **Comparison *+* vs *- nhsf-1*** | **ratio *+/- nhsf-1* in WT vs condition** | **ratio *+/- nhsf-1* in tax-4(p678) vs condition** | **Neuron(s) involved?** |
| WT +/- *hsf-1^neuro^* | *fat-7p::GFP*  Fig 3I-K | negative control | 31.9% decrease | _ | 0.0077 (**) | *_* |
| *tax-4(p678)lof* *+/- hsf-1^neuro^* |  | positive control | 19.5% decrease | 0.0029 (**) | _ | *_* |
| *tax-4(p678)lof* *+/- hsf-1^neuro^* |  | tax-4(+) in **ASG** *(pops-1p)* | 9.3% decrease | 0.0004 (***) | 0.1585 (ns) | no |
| *tax-4(p678)lof* *+/- hsf-1^neuro^* |  | tax-4(+) in **ASJ** *(trx-1p)* | 5.3% decrease | <0.0001 (****) | 0.0123 (*) | no |
| *tax-4(p678)lof* *+/- hsf-1^neuro^* |  | tax-4(+) in **ASI** *(str-3p)* | 18% decrease | 0.0094 (**) | 0.7949 (ns) | no |
| *tax-4(p678)lof* *+/- hsf-1^neuro^* |  | tax-4(+) in **AWC** *(ceh-36p)* | 16.2% decrease | 0.0094 (**) | 0.7949 (ns) | no |
| *tax-4(p678)lof* *+/- hsf-1^neuro^* |  | tax-4(+) in **ASJ**+**ASI** | 10.3% decrease | 0.0005 (***) | 0.1759 (ns) | no |
| *tax-4(p678)lof* *+/- hsf-1^neuro^* |  | tax-4(+) in **ASJ**+**AWC** | 5.9% decrease | <0.0001 (****) | 0.0009 (***) | no |
| *tax-4(p678)lof* *+/- hsf-1^neuro^* |  | tax-4(+) in **AWC**+**ASI** | 8.6% decrease | 0.0004 (***) | 0.1585 (ns) | no |
| *tax-4(p678)lof* *+/- hsf-1^neuro^* |  | tax-4(+) in **AW**C+**ASI**+**ASJ** | 12.6% decrease | 0.0021 (**) | 0.3971 (ns) | no |
| **Genotype** | **Assay** | **neuron(s) tested** | **Comparison *+* vs *- nhsf-1*** | **Significant?** | | **Neuron(s) involved?** |
| WT *+/- hsf-1^neuro^* | *fat-7p::GFP* Fig 3H | controls | 34% decrease | 2way ANOVA interaction:  Similar effect of *hsf-1^neuro^* across genotypes: p=0.6386 (ns) | | _ |
| *ttx-1(p767)lof* *+/- hsf-1^neuro^* |  | **AFD** genetic ablation | 33% decrease |  |  | no |
| WT +/- *hsf-1^neuro^* | *lipl-1, lipl-3* qRT-PCR Fig 3I-K | negative control | *lipl-1:* 11.4 fold, *lipl-3:*6.1 fold | *nhsf-1* effect differs across genotypes  p<0.0001 (*lipl-1*) 2way ANOVA*,  p<0.0001 (*lipl-3*) mixed effect analysis* | | *_* |
| *tax-2(p671)lof* *+/- hsf-1^neuro^* |  | positive control | *lipl-1*: 0.3 fold, *lipl-3*: 0.5 fold |  |  | *_* |
| WT +/- *hsf-1^neuro^* |  | negative control (exp ASJ,AWB,ASK) | *lipl-1*: 2.3 fold, *lipl-3*: 3.5 fold | 2way ANOVA interaction*:  Similar effect of *hsf-1^neuro^* across genotypes:  p=0.8221 (*lipl-1*) p=0.9936 (*lipl-3*) | |  |
| ASJ(-) +/- *hsf-1^neuro^* |  | **ASJ** genetic ablation (*trx-1p::TeTx*) | *lipl-1*: 16.1 fold, *lipl-3*:5.3 fold |  |  | no |
| AWB(-) +/- *hsf-1^neuro^* |  | **AWB** genetic ablation (*str-1p::casp-1*) | *lipl-1*: 3.6 fold, *lipl-3*: 2.4 fold |  |  | no |
| ASK(-) +/- *hsf-1^neuro^* |  | **ASK** genetic ablation (*sra-9p::casp-1*) | *lipl-1*: 4.0 fold, *lipl-3*: 2.1 fold |  |  | no |

*statistical analysis was performed on log-transformed data as range of data differed with the scale – *lof*: loss of function

**S9 Table.** **The activation of *hsf-1^neuro^* in six or more *tax-2/tax-4* expressing neurons is required for remote fat remodelling.** Related to Figure 3F-K.

Rescue in *tax-2(p694)* allele which eliminates the function of neurons indicated in **Fig 3I**, suggesting that  *hsf-1^neuro^* expression in specific or a combination of ASG, ASJ, ASK, AWB, ASI, AWC neurons must be responsible for controlling fat accumulation. **Fig 3J** shows a diagram summarizing all the experiments performed to genetically dissect individual or combinations of neurons that mediate  *hsf-1^neuro^* dependent fat remodelling. We used different techniques to test specific neuron(s) involvement in  *hsf-1^neuro^*  dependent lean phenotype. Tax-4 rescue assays*: tax-4(+)* was expressed in specific neurons in *tax-4(p678)* null background (red neurons in **Fig 3J**). The expression of *tax-4* cDNA was controlled by the following neuron-specific promoters: *pops-1p* for ASG, *trx-1p* for ASJ, *ceh-36p* for AWC, and *str-3p* for ASI. Expression in multiple neurons was achieved by co-expressing several neurons specific *tax-4(+)* rescue constructs. In vivo fluorescent measurement of *fat-7pgfp* levels in worms carrying  *hsf-1^neuro^* were monitored to check  *hsf-1^neuro^* effect on fat remodelling. Removal of AFD function (orange neuron in **Fig 3J**): TTX-1 is an otd/otx homeodomain transcription factor expressed solely in the AFD thermosensory neuron, and which is required for the specification of the AFD neuron (42). Mutants carrying *ttx-1(p767)* exhibit thermotactic behavioural defects phenocopying AFD ablation (47). Genetic ablation and qRT-PCR assays: When *fat-7p::gfp* reporter could not be used, mRNA levels of *lipl-1* and *lipl-3* mRNA were monitored by qRT-PCR (blue and purple neurons in **Fig 3J**) in worms carrying genetic ablation of neurons of interest. Genetic ablation was obtained either by AWB and ASK specific expression of caspase 1, using the promoters *str-1p* and *sra-9p*, respectively, or by ASJ specific expression of tetanus toxin using the *trx-1p* promoter. Tetanus toxin (TeTx) prevents neurotransmission from the neuron it is expressed in. No single *tax-2/4* expressing neuron is responsible for  *hsf-1^neuro^* dependent fat remodelling. Combinations of two or three neurons (ASJ+ASI; ASJ+AWC; ASI+AWC+ASJ) were also found ineffective at suppressing tax-4 loss of function. However, as described in **Fig 3F**, six *tax-2/tax-4* expressing neurons are sufficient to partially rescue  *hsf-1^neuro^* phenotype. Because *tax-2(p694)* only achieves partial rescue, we conclude that the activation of stress responses in six or more TAX-2/4 sensory neurons is required to change fat metabolism.
