# Supplementary material for: Neuronal HSF-1 coordinates the propagation of fat desaturation across tissues to enable adaptation to high temperatures in C. elegans
Source: PLoS Biol. 2021 Nov 1;19(11):e3001431. doi: 10.1371/journal.pbio.3001431 (PMC8585009; doi:10.1371/journal.pbio.3001431)
Supplement: S10 Table — (DOCX) [file pbio.3001431.s019.docx]

**S10 Table: Fertility assay,** related to Figure S3B.

| **Strain** | **Condition** | **Average** | **N value** | **Number of Biological Replicates** | **P value (vs control)** | **Statistical test** |
| --- | --- | --- | --- | --- | --- | --- |
| N2/CTR | N2 day 1 | 47.30 (4.49) | 62 | 3 |  | 2 way ANOVA (days 1 to 5) |
| N2/CTR | N2 day 2 | 125.60 (20.86) | 60 | 3 |  | Effect of Genotype: P-value=0.125 (ns) |
| N2/CTR | N2 day 3 | 87.03 (14.47) | 59 | 3 |  | Effect of Day: P-value <0.0001 (***) |
| N2/CTR | N2 day 4 | 9.50 (0.90) | 59 | 3 |  | Genotype x day,  P-value= 0.0293 (*) |
| N2/CTR | N2 day 5 | 1.97 (1.53) | 56 | 3 |  |  |
| AGD1289  (*hsf-1^neuro^#2*) | AGD1289 day 1 | 35.64 (6.21) | 49 | 3 | P=0.9 vs N2 day 1 | Positive 2-way ANOVA interaction |
| AGD1289  *hsf-1^neuro^#2* | AGD1289 day 2 | 55.31 (18.07) | 47 | 3 | P=0.04 vs N2 day 2 | Positive 2-way ANOVA interaction |
| AGD1289  *hsf-1^neuro^#2* | AGD1289 day 3 | 40.99 (8.25) | 48 | 3 | P=0.04 vs N2 day 3 | Positive 2-way ANOVA interaction |
| AGD1289  *hsf-1^neuro^#2* | AGD1289 day 4 | 19.10 (1.84) | 48 | 3 | P=0.9 vs N2 day 4 | Positive 2-way ANOVA interaction |
| AGD1289  *hsf-1^neuro^#2* | AGD1289 day 5 | 11.48 (2.33) | 46 | 3 | P=0.9 vs N2 day 5 | Positive 2-way ANOVA interaction |
| **Strain** | **Condition** | **Total nb of progeny (+/- SEM)** | **N value** | **Number of Biological Replicates** | **% of CTR** | **Statistical test** |
| N2/CTR | day-day5 | 271.4 (29.27) | 296 | 3 |  | paired t-test (vs CTR) |
| AGD1289  *hsf-1^neuro^#2* | day1-day5 | 172.1 (19.36) | 238 | 3 | 37% less | 0.1078 (ns) |
