# Supplementary material for: Neuronal HSF-1 coordinates the propagation of fat desaturation across tissues to enable adaptation to high temperatures in C. elegans
Source: PLoS Biol. 2021 Nov 1;19(11):e3001431. doi: 10.1371/journal.pbio.3001431 (PMC8585009; doi:10.1371/journal.pbio.3001431)
Supplement: S11 Table — (DOCX) [file pbio.3001431.s020.docx]

**S11 Table – Number of GFP positive neurons in PS7171 (*hsp-16.41*p::cGAL/UAS::GFP) at different growth temperatures.** Related to Figure 6.

| **Condition** | **Average nb GFP+ neurons per worm (+/- SEM)** | **n- value** | **BR** | **Comparison** | **Fold Change** | **P-value** | **Statistical Test** |
| --- | --- | --- | --- | --- | --- | --- | --- |
| PS7171 15 °C | 0.39 (0.1) | 105 | 3 | 15 °C vs 20 °C | 6.6 up at 20°C | ns | RM-One way ANOVA |
| PS7171 20 °C | 2.6 (0.5) | 215 | 3 | 20 °C vs 25 °C | 2.5 up at 25°C | 0.0174 | Tukey’s test |
| PS7171 25 °C | 6.4 (0.99) | 254 | 3 | 15 °C vs 25 °C | 16 up at 25°C | 0.0032 |  |

BR: Biological replicates
