# Supplementary material for: Neuronal HSF-1 coordinates the propagation of fat desaturation across tissues to enable adaptation to high temperatures in C. elegans
Source: PLoS Biol. 2021 Nov 1;19(11):e3001431. doi: 10.1371/journal.pbio.3001431 (PMC8585009; doi:10.1371/journal.pbio.3001431)
Supplement: S12 Table — (DOCX) [file pbio.3001431.s021.docx]

**S12 Table: Primers used in this study**

| **Genotyping primers** | | |  |  |  |  |
| --- | --- | --- | --- | --- | --- | --- |
| **Allele** | **Identifier** | **Sequence (5' to 3')** | **Identifier** | **Sequence (5' to 3')** | **mutation type** | **length amplified** |
| *dbl-1(nk3)* | dbl-1 EXT F | TCTGCATCGTATCAGTTCCTC | dbl-1 EXT R | CGGTTGAAGGTGAGAAATGAGG | 5690 bp deletion (Morita 1999) | WT: 5926 bp, dbl-1(nk3): 331 bp |
| *dbl-1(nk3)* | dbl-1 EXT F | TCTGCATCGTATCAGTTCCTC | dbl-1 INT R | TCCTGTACTTTACTCGTCATGG | 5690 bp deletion (Morita 1999) | WT: 689 bp, dbl-1(nk3): 0 bp |
| *rrf-3(pk1426)* | rrf-3 EXT F | ACGTAATAGAATACTCATCCGCT | rrf-3 EXT R | GCAGCATGTCCAGACACAAC | 3015 bp deletion | WT: 3483 bp, rrf-3(pk1426): 468 bp |
| *rrf-3(pk1426)* | rrf-3 INT F | TCGCACAAACTTGGCAATCG | rrf-3 INT R | TTCAAGGAACATACTGGAAAAGTC | 3015 bp deletion | WT: 659 bp, rrf-3(pk1426): 0 bp |
| *tax-2(p671)* | tax-2(p671) F | TCGACCGAGTTTGATGTCATTG | tax-2(p671) R | TAACCGTGGTTTGATTAGCAGC | t>c in tax-2(p671) at nt 204 of PCR | 347 bp to be sequenced |
| *tax-2(p694)* | tax-2(p694) F | AAGAGGCTATTCGAGCACACG | tax-2(p694) R | TCGGCAAATCGTTTCACAACT | 354 bp indel (Bretscher, 2011). | WT:1335 bp, tax-2(p694): 945bp |
| *tax-4(p678)* | tax-4(p678) F | TACGACTACGGCTCAGCAAA | tax-4(p678) R | AGAGGCACACATTCTTTTCCA | c>t in tax-4(p678) at nt 190 of PCR | 424 bp to be sequenced |
| *ttx-1(p767)* | ttx-1(p767) F | ACAAATGCTAAGCTTTCAAATGCG | ttx-1(p767) R | GCTGAGCCAGATGATTGGGA | g>a in ttx-1(p767) at nt 599 of PCR | 771 bp to be sequenced |
| *ttx-3(ks5)* | ttx-3(ks5) F | AAATGCGAAATCCCATTGAA | ttx-3(ks5) R | AAATGACCTTGCGGAGACAT | g>a in ttx-3(ks5) at nt 85 of PCR | 200 bp to be sequenced |
| **qRT PCR primers** | |  |  |  |  |  |
| **mRNA target** |  | **Forward** |  | **Forward** | **PCR efficiency** | **R^2** |
| *cdc-42* | *cdc-42 F* | TCCACAGACCGACGTGTTTC | *cdc-42 R* | AGGCACCCATTTTTCTCGGA | 100.3 | 0.99 |
| *gfp* | *gfp F* | TGTTCCATGGCCAACACTTG | *gfp R* | CCTGTACATAACCTTCGGGCA | 99.1 | 0.99 |
| *ire-1* | *ire-1 F* | TACTTGCCACCACGGAGACC | *ire-1 R* | CGTTGCCATCGTCATCATTG | 110.3 | 0.99 |
| *lipl-1* | *lipl-1 F* | ATCGGTTTGCGCTGGACTTA | *lipl-1 R* | CACGAGTTGCGTTAAGCTGG | 103.9 | 0.99 |
| *lipl-2* | *lipl-2 For* | ATGGACAGGCTAATCCCCCA | *lipl-2 R* | ACCAATCGGCATCACTCCAG |  |  |
| *lipl-3* | *lipl-3 For* | GCTGGTACTGCCACACAGAA | *lipl-3 R* | GGTGGATTTGCCTGCCCATA | 106.1 | 0.99 |
| *mir-80* | *mir-80 For* | ATGGACACTCGTTCGCTCAG | *mir-80 R* | AATGATCTCAACAACATGGGCT | 112.5 | 0.99 |
| *pmp-3* | *pmp-3 For* | GTTCCCGTGTTCATCACTCAT | *pmp-3 R* | ACACCGTCGAGAAGCTGTAGA | 109.3 | 0.99 |
