# Supplementary material for: Neuronal HSF-1 coordinates the propagation of fat desaturation across tissues to enable adaptation to high temperatures in C. elegans
Source: PLoS Biol. 2021 Nov 1;19(11):e3001431. doi: 10.1371/journal.pbio.3001431 (PMC8585009; doi:10.1371/journal.pbio.3001431)
Supplement: Attachment — Submitted filename: Response to Reviewers.docx [file pbio.3001431.s023.docx]

REVIEWS:

Reviewer #1: Using a C. elegans strain over-expressing hsf-1 in neurons, the authors of the manuscript comprehensively characterized phenotypic consequences of fat remodelling in non-neuronal peripheral tissues. Based mainly on these findings and activation of HSF-1 target gene reporters in neurons by chronic warmer temperature, the authors conclude that HSF-1 in neurons serve as a thermostat-based mechanism to cell non-autonomously coordinate membrane saturation and composition across tissues in a multicellular animal. While the observations are interesting, there are major issues of this paper that need to be addressed before suitable publication.

We thank our reviewer for their thoughtful comments on the manuscript.

**1, Almost the entire paper is based on studies of the C. elegans strain over-expressing hsf-1 in neurons. How do conclusions from such studies say anything about the physiological roles of endogenous HSF-1 in neurons? Does loss of function of hsf-1 in neurons (by either Cre-LoxP deletion or neuronal rescued of hsf-1 KO, both are technically feasible) have any phenotypes in fat remodelling of peripheral tissues? Activation of HSF-1 target gene reporters in neurons by chronic warmer temperature is evidence of correlation in nature, providing no causal relationship between neuronal HSF-1 and fat remodelling in peripheral tissues.**

We thank the reviewer for highlighting the importance of showing that neuronal *hsf-1* is needed under physiological conditions. We have provided several lines of evidence to further strengthen this point.

First, we have knocked down the expression of *hsf-1* using a pan-neuronal RNAi sensitive strain (TU3401*:sid-1(pk3321)V,uIs69 [pCFJ190(myo-2p::mCherry*) + *unc-119p*::sid-1) (1). In **Figure 5 F-I** and **Table S8**, we show that knock down of *hsf-1* in neurons causes the expected impairment in heat shock response activation. In animals fed with empty vector, acid lipases are up-regulated in worms that are raised at 25°C (**Figure 1, Figure 5 F-G**), this upregulation is prevented in animals fed with *hsf-1* RNAi.

Recently, Omi and Pujol (2) reported that *unc-119* promoter has some leaky activity in the hypodermis. To control for a potential spurious effect, we used the strain QK52: *rde-1(n219); xkls99[wrt-2p::red-1::unc-543’UTR]*, where *wrt-2p* is a *bona fide* hypodermis promoter. We found that although *hsf-1* KD in the epidermis causes an impairment in the heat shock response, it does not have any detectable effect on lipase expression (**Figure S8**, also added below).

These experiments indicate that while RNAi treatment is effective in both strains, *hsf-1* in neurons -and not in the hypodermis- is necessary for lipase expression. Together with results shown in **Figure 1**, we can confidently say that pan-neuronal expression of *hsf-1* is both necessary and sufficient to ensure that acid lipases (*lipl-1-3*) are expressed in the gut when HSF-1 is active in neurons.

**Figure S8. Neuronal and not epidermis expression of *hsf-1* is necessary for lipase expression**

**At 25°C.** Relative expression levels of *hsp16.1, hsp16.41*, *lipl-1* and *lipl-2* mRNA by qRT-PCR Two strains were used: TU3401*(sid-1(pk3321)V,uIs69 [pCFJ190(myo-2p::mCherry*) + *unc-119p*::*sid-1*) which is primarily a pan neuronal RNAi sensitive strain with some activity in the epidermis and QK52: *rde-1(n219); xkls99[wrt-2p::red-1::unc-543’UTR]* which is an epidermis-specific RNAi sensitive strain. Animals were raised at 25°C for 1 generation and were fed either L4440 (empty vector, EV) control or *hsf-1*double stranded RNA (Ahringer RNAi library) for 48h, from late L4 onwards. To ascertain the effectiveness of the RNAi treatment we looked at the effect of *hsf-1* KD on the heat shock response. Animals were placed under heat shock conditions and the expression of chaperones (*hsp16.1* and *hsp16.41)* were tested 30 minutes after the heat shock (see materials and methods for details). As expected. KD of *hsf-1* in both neurons and epidermis causes a dampening in the heat shock response. Levels of mRNA levels were measured at day 3 of adulthood. Statistics were performed using paired t-test (**S8 Table**). Bars represent the SEM and each dot represents a biological replicate.

**Figure 5 H-I** shows that knocking down *hsf-1* from neurons does not change the expected decrease in *fat-6* and *fat-7* at 25°C, although **Figure 1** shows that neuronal over-expression of *hsf-1* is sufficient to downregulate their expression at 20°C. These results indicate that pan-neuronal *hsf-1* is sufficient but not necessary to regulate *fat-6/7* expression. We propose that in relation to fat desaturases *hsf-1^neuro^* downregulates BMP signalling, but that other redundant signals are required at 25°C to further control them under physiological conditions (**Figure 4H, Figure 6** and **Figure S9**).

We also show that the gene expression changes have functional consequences for survival. Specifically, we show in **Figure 5K** and **Supplementary Table 12**, that the K.O. of *hsf-1* using the aforementioned RNAi sensitive strain causes worms to survive significantly less than control animals at 25°C, We believe that these results are a strong indication that neuronal HSF-1 plays a significant role under physiological conditions and have added them to the main text.

**2, Technically, how do authors exclude the possibilities that the so-called neuronal rab-3 promoter does not have residual activity in non-neuronal tissues responsible for the phenotypes observed? This is particularly a concern when high-copy number instead of single-copy transgene was used in this case, and the conclusion is based on a single "neuronal specific" promoter in an overexpression system.**

The *rab-3* promoter has been widely used in the *C. elegans* neuroscience community as a reliably pan-neuronal promoter when expressed from multi-copy extra-chromosomal arrays. Papers that have used the *rab-3* promoter in this way include Stefanakis *et* al, 2015 in Neuron (3) and Nguyen *et* al 2016 in PNAS (4), among others. Searching the expression of *rab-3* using the standard *GExplore 1.4* tool (5), which is based on single-cell gene expression data and the *Tomoseq* tool, which shows high-resolution gene expression maps obtained from cryo-sections of *C. elegans* (6) shows that *rab-3* is expressed at significant levels in neuronal tissues only. Gene expression driven by the *rab-3* promoter from transgenes has been also shown to be specific to neurons (3). Together, these data, and the extensive use of the *rab-3* promoter for similar purposes, suggest it does not have significant residual activity in other tissues.

**3, The authors used RAD-SMAD reporters to monitor regulation of BMP by HSF. This should be complemented with studies (QPCR and low-copy transgene etc) on the regulation of specific and endogenous BMP pathway genes (e.g. dbl-1) to convincingly support the model proposed. More importantly, it remains unclear whether such potential regulation of BMP/dbl-1 by HSF-1 actually contributes to fat remodelling. Can transgenic rab-3p::dbl-1 in hsf-1neural worms restore fat-7 expression and lipid saturation profiles?**

To complement our use of RAD-SMAD reporters to monitor BMP activity, we have used extensive genetic epistasis analyses (**Figure 5**). All of these analyses showed that SMA/BMP signalling is epistatic to *hsf-1^neuro^* for *fat-6/7* expression, lipid droplet accumulation and small body size. It is however not epistatic with regards to the expression of acid lipases, indicating that BMP is a signal that modulates the expression of only one of the signalling branches controlled by *hsf-1^neuro^* (see **Figure 6** for clarification).

In response to this point of the reviewer, we have performed additional experiments to further strengthen this link. First, in **Figure S5A,** we have addressed if *hsf-1^neuro^* changes the expression of *dbl-1* and of downstream signal transduction components of the SMA/BMP pathway. We have found that this is indeed the case, as the transcripts of *dbl-1*, *sma-3(R-SMAD)*, *sma-4 (Co-SMAD)* and *sma-6* -the later encoding a Type-I receptor of TGF-β/BMP signalling- are all downregulated when HSF-1 is over-expressed in neurons. We have added these results to the main text (lines 467-474). A reduction in transcripts could be caused by controlling the transcriptional output driven by their promoters or by controlling the stability of the transcript post-transcriptionally.

To start addressing this issue we looked at a transcriptional reporter of *dbl-1,* which is expressed primarily in ventral cord neurons and can be visualised using a transcriptional reporter that carries a heterologous 3’UTR (*ctIs43[dbl-1p::GFP+dbl-1p::GFP::NLS]* (6)). We observed that *hsf-1^neuro^* causes a downregulation in GFP expressed from this reporter (quite evident in **S5 B-D),** however the difference is not significant. We therefore conclude that regulation of *dbl-1* by *hsf-1^neuro^* is likely to be influenced by post-transcriptional mechanisms (main text, lines 471-4).

With regards to the second point raised by the reviewer. **Can transgenic rab-3p::dbl-1 in hsf-1 neural worms restore fat-7 expression and lipid saturation profiles?**

We agree with reviewer 1, that if there was a linear relationship between neuronal *hsf-1* and BMP signalling the expectation is that the overexpression of *dbl-1* should rescue fat remodelling in *hsf-1^neuro^*. However, overexpression of *dbl-1* (using *dbl-1p::dbl-1::TM::mcherry,* (8)) behaves in the same manner as the loss of *dbl-1* function, to decrease *fat-7p::GFP* levels as well as fat accumulation (**Figure S5E**). We know that the transgene is functional because animals show a long phenotype and others have also observed a decrease in *fat-7* expression, comparable to *dbl-1* mutants (9). Although initially these results are surprising, we believe they are consistent with the lack of “necessity” for neuronal *hsf-1* in the control *fat-6/7* expression. We propose that BMP has to be present at the correct dose in order for other -unknown- instructive signals to upregulate *fat-6/7* expression. We have added these results (lines 457-462) and summarised this model in **Figure 6**.

**4, Recent studies have demonstrated importance of several "cold sensors" in C. elegans involved in neuronal sensing of cold temperature and peripheral tissue lipid homeostasis. It would be important to test if those pathways (trpa-1 and paqr-2) act independently with HSF-1 or not in lipid remodelling.**

We agree with the reviewer that this line of enquiry is important, however we consider it beyond the scope of this work.

Reviewer #2: In this important manuscript, Chauve and colleagues report the finding of a thermostat that cell non-autonomously coordinates global changes in membrane composition and metabolism allowing an ectotherm to adapt to higher temperatures and enhance survival in these conditions. 
The authors show that C. elegans worms that express hsf-1 pan-neuronally display transcriptomic and metabolic profiles similar to those displayed by animals grown at 25 ºC despite having grown at 15 ºC. This is a shift in fat desaturases and membrane lipid composition that requires the function of cGMP gated-channels tax-2 and tax-4, which are expressed in sensory neurons. Then, they go on to identify a group of six sensory neurons where tax-2 function is sufficient to drive the nhfs-1-associated changes in lipid composition. Finally, they identify the suppression of TGF beta signalling as one of the mechanisms by which the effects of nhfs-1 are implemented. 
In my opinion, the finding reported here is extremely novel and it changes the way we thought about how ectotherms regulate temperature-associated adaptive changes. The data has been carefully acquired, supports the conclusions stated and allows the authors to generate a model that, even if it may be incomplete for the time being since this is a novel finding, is informative and useful.

**I only have one comment to make regarding the data of tax-4 presented in page 20 and figure 3C. The authors mention that disruption of tax-4 (in p678) "does not significantly alter the output of fat-7::gfp reporter in wildtype animals" but there is no statistical analysis for this comparison.**

Thanks for pointing this out. We have tried to simplify figures by only showing significant p values. In **Figure 3C**, the reviewer is right to point out that in *tax-4* mutants the baseline *fat-7p:gfp* expression is higher than wild types. In **Table S6**, the mean intensity values are: *tax-4(+)* =11619(+/-867SEM), n=233 versus *tax-4(p678)=*14924(+/-1206SEM), n=224.

However, we find that this difference is not statistically significant. We feel that this data is quite robust, as we took 7 biological replicates to make this point and the technique to measure GFP fluorescence is not prone to overt batch-to-batch variability. We have added this point to the figure legends, to the figure itself and the main text (line 340).

**They also say that the effect of hsf-1neural on lipid enzymes is dependent on tax-4 and that both genes act in a linear pathway. However, hsf-1neural can still significantly reduce fat-7::gfp levels in tax-4 mutants (figure C) and so, the interpretation is that hsf-1neural acts through tax-4 dependent and tax-4 independent pathways.** 

The reviewer is correct, *tax-4(p678)* mutants rescue most but not all of the *hsf-1^neuro^* related *fat-7* expression (**Figure 4C**). *Tax-2 (p671),* however, rescues both the lipid droplet accumulation (**Figure 4F**) and the lipase expression phenotypes almost completely (**Fig 4 D** and **E**). We cannot easily perform the epistasis experiment between the *fat-7* reporter and *tax-2*, because they are linked on the same chromosome. However, we noticed in new lifespan data, that *tax-2(p671*) does not completely abrogates the advantage of *hsf-1^neuro^* at 25°C (**Figure S2**).

We agree with the reviewer that although *tax-2* causes substantial suppression of *hsf-1^neuro^* there may be a small effect which is independent of TAX-2/4 function. An alternative explanation is that both alleles used in the study cause strong loss of function phenotypes, but may not be complete nulls. While the *p671* allele is a C231R missense mutation, the *p678* allele of *tax-4* is a Q to amber stop substitution in the 82th amino-acid. Therefore, although both are considered to be null alleles, there could be some residual/ temperature sensitive activity. We have explicitly highlighted the point raised by our reviewer in the main text (line 357 onwards) and legends.

Reviewer#3: 
The authors present impressive amount of data to show that neuronal HSF-1 signalling co-ordinately remodels lipid composition across tissues to adapt to thermal variation. The authors clearly show that overexpression HSF-1 in neurons induces lipid desaturation in a cell non-autonomous manner. The effect of neuronal HSF-1 expression on fat desaturation requires (at least in part) the cyclic nucleotide gated cation channel TAX-2/TAX-4 in sensory neurons results in downregulation of TGF-β/BMP signalling to modulate fat metabolism. This work suggests a novel mechanism whereby lipid composition can be centrally regulated in a multicellular organism to facilitate membrane stability over a wide range of temperatures. This is an interesting and novel finding. However, I have some concerns to what extent the considerable amount of data support their model.

We thank our reviewer for the thoughtful comments on the manuscript.
Major points:
**1.      The authors present a model in which hsf-1 acts in the TAX-2/TAX-4 sensory neurons as a thermostat to control lipid remodelling and heat adaptation. However, it is not clear whether hsf-1 expression in TAX-2/TAX-4 sensory neurons is sufficient for lipid remodelling and an increase lifespan at 25C. They should strengthen the argument by testing if the expression of hsf-1 specifically in those neurons (e.g. tax-2::hsf-1) is sufficient to control fat metabolism. The argument also should be strengthened by checking if HSR is activated in those six neurons at 25°C (e.g. hsp-16.41::GFP expression, hsf-1 expression, or hsf-1 nuclear localization).**

We thank the reviewer for raising the important point of whether the relationship between *hsf-1^neuro^* and *tax-4* is direct or indirect. We have addressed these points by expressing *hsf-1* from *tax-4* promoter (**Figure S3**). We find that over-expression of *hsf-1* from a *tax-4* promoter recapitulates the effect of pan-neuronal (*rab-3*-driven) overexpression for *fat-7P:gfp* expression but not for *lipase1-3* expression (lines 362 onwards in the main text).

We have also performed co-localisation experiments in wild type animals carrying two transgenes. The first one is a UAS/Gal-4 binary system that allow detection of GFP driven by an HSF-1 target: the *hsp-16.41* promoter. Using this reagent, GFP -which indicates an active heat shock response (HSR), is evident primarily in head neurons at physiological temperatures. The same strain carried a transgene that allows detection of *tax-4* expressing neurons (*tax-4p::tax-4::wrmScarlet* in **Figure S6A** and **movie 1** and *tax-4p::his-24::mOrange* in **Figure S6B** and **movie 2**)*.* The analysis of both reporters show that no direct co-localization is evident between neurons expressing *tax-4* and neurons experiencing a heat shock response (HSR). We have added the results to the main text (line 499 onwards). In addition, we present a revised model summarized below.

We have discovered a previously unknown and fundamental neuro-endocrine pathway that links neuronal stress activation with a fat remodelling program that contributes to membrane acclimation and survival at warm temperatures. We propose that a thermostat switches on this program when temperatures rise above 20°C. We highlight multiple aspects of the underlying control mechanisms, but also find that, perhaps unsurprisingly, the mechanism is complex. We have simplified the model so readers can capture the logic of the system despite its complexity.

Our results implicate two distinct neuro-endocrine pathways and a complex circuitry. One pathway, mediated via BMP/SMA signalling, is responsible for adjusting fat desaturase expression in the gut. Another pathway, -controlled by an unknown molecular signal- adjusts acid lipases expression in the gut.

We have summarised the model in a diagram (**Figure 6**). Below is the evidence we have accumulated supporting this model.

**Lipase branch:**

We find that both over-expression and knock down of pan-neuronal *hsf-1* alters expression of acid lipases ((*lipl-1* to *lipl-3*, **Figures** **2D and 5F-G**). These results indicate that *hsf-1* activity is both necessary and sufficient in head neurons to activate acid-lipase expression in the gut.

We also find that disrupting *tax-2/4* function can suppress the effect of pan-neuronal *hsf-1* over-expression on lipase expression (**Figure 3 D-E**). One potential model would be that *hsf-1* acts directly in *tax-2/4* expressing neurons to exert this effect. However, we find that when *hsf-1* is expressed only from the *tax-4* promoter, *lipase 1/3* expression is not upregulated in the gut (**Figure S3**), suggesting that the role of *tax-2/4* neurons is indirect. Consistent with this idea, we find that although occasionally *tax-2/4* neurons can activate the HSR (**Figure S6B, movie_2)**, the neuronal induction of HSR at 25°C does not occur frequently in *tax-4* expressing neurons (**Figure S6A**). However, many other neurons consistently upregulate HSR (**Figure S6A)**. These results support a model where TAX-2/4 neurons have an indirect effect on the “thermostat” that controls acid lipases and future work should identify these neurons.

**Fat desaturase branch:**

For the neuroendocrine signalling pathway that controls *fat-6/7*, the picture is different. We propose the following model. Over-expressing *hsf-1* pan-neuronally (**Figure** **1 H-K**) or from the *tax-4* promoter (**Figure S3**), downregulates *fat-7* expression. Moreover, removing *tax-4* or *tax-2* function suppresses *hsf-1*-driven inhibition of *fat-6/7* expression, (**Figure 3A-C**). These observations are consistent with a model in which activating *hsf-1* in *tax-2/4* expressing neurons is able to control expression of fat-desaturases in the gut. However, removing *hsf-1* function from all head neurons (**Figure 5 H-I**) does not change *fat-7* levels. These results suggest that while *hsf-1* activity in TAX-2/4-expressing neurons is sufficient to modulate fat desaturase expression, it is not necessary for this effect.

The results suggest that additional pathways besides *hsf-1* act in wild type animals to ensure gut expression of *fat-7* when neuronal *hsf-1* activity decreases. We have observed that neuronal *hsf-1* activity is reduced by exposure to cold temperatures (**Figure 5A-B**). However, our results suggest that cold-regulated genes (such as *fat-7*) are not activated simply by a decrease in neuronal *hsf-1* function, and that other instructive programs must be in place to regulate their expression. In fact, we find that over-expressing *dbl-1* is not sufficient to up-regulate *fat-7* expression (**Figure S6** and (8), suggesting that additional molecular programs must be in place to ensure survival in the cold. Many potentially relevant pathways have been previously described in the literature and additional work is needed to disentangle this circuitry.

We have summarised this model in **Figure 6** and have extensively changed the main text and discussion to reflect our new findings.

**2.      The authors claim that overexpression of hsf-1 in neurons suppresses TGF-β/BMP signalling to remodel fat metabolism. Currently the link between HSF-1 and TGF-β/BMP signalling is tentative. The function of TGF-β/BMP signalling in regulating lipid metabolism including fat-6/fat-7 expression is well known (Luo et al., 2010; Yu et al., 2017; Clark et al., 2018). What remains unknown is how HSF-1 functions to reduce TGF-β/BMP signalling. dbl-1 is expressed mainly in neurons. It would be interesting to test if expression of dbl-1 in neurons (also in tax-2/tax-4 neurons?) is decreased at 25ºC and in the hsf-1neural animals even at 20ºC.**

We agree with the reviewer’s comment, and have done several lines of experiments to address the point s/her raises.

First, we used qPCR to determine if transcript levels for *dbl-1* or its signal transduction components in the BMP/SMA pathway are changed upon overexpression of *hsf-1* in neurons at 20°C. In **Figure S5A** we show that *hsf-1^neuro^* is able to transcriptionally downregulate the transcripts of *dbl-1* as well as of several signal transduction components of BMP/SMA pathway, including *Sma-3(R-SMAD)* and *sma-4 (Co-SMAD)* which encode intracellular signalling transducers of TGF-β/BMP signalling pathway, and *sma-6* -encoding a Type-I receptor of TGF-β/BMP signalling. The results have been added in the text from line 466 onwards.

We also quantified fluorescence from the *ctIs43[dbl-1p::GFP+dbl-1p::GFP::NLS]* transcriptional reporter (7) in wild type and *hsf-1^neuro^* background (**Figure S5 B-D**). The reporter is expressed in the ventral cord nerve (VCN) and shows a clear pattern of downregulation, although due to variability in the assay, it did not reach significance. We suggest that regulation of BMP related transcripts is post-transcriptional and have added to the main text (lines 471-4).

Our conclusion -based on the RADSMAD reporter (**Figure 4A-C**), the results from genetic epistasis experiments (**Figure 4D-G**) and the new results presented in **Figure S5**- is that overexpressing *hsf-1* in neurons is sufficient to downregulate BMP/SMA signalling by decreasing expression of the ligand *dbl-1* and downregulating the expression of several signal transduction components of the BMP/SMA pathway.

**It would be interesting to test if expression of dbl-1 in neurons (also in tax-2/tax-4 neurons?) is decreased at 25ºC and in the hsf-1neural animals even at 20ºC.**

Due to limited time, we were unable to perform experiments to address this point.

**Their argument could be strengthened by testing if overexpression of dbl-1 is able to rescue the hsf-1neural phenotypes and if dbl-1 expression in neurons and TGF-β/BMP activity in intestine (RAD-SMAD reporter) is increased in hsf-1 (sy441 and/or RNAi) mutants at 25ºC.**

We appreciate that this is an important set of experiments. *dbl-1*(lof) animals are small whereas *dbl-1*(gof) are long. One would therefore expect that *dbl-1*(lof) and *dbl-1*(gof) should have opposite phenotypes in terms of their fat accumulation profiles. However, Clark and colleagues (9) observed that is not the case: long (*dbl-1*-gof) animals are as lean as short (*dbl-1*-lof) animals. We have recapitulated these observations in **Figure S5E**. We used a transgene that overexpresses of *dbl-1* from its own promoter (using *dbl-1p::dbl-1::TM::mcherry*) which causes worms to be longer (8). Animals carrying the transgene do not rescue *hsf-1^neuro^* levels of *fat-7p::GFP* expression. The results are described in the main text lines 458-462.

BMP signalling has long been recognised as a dose-dependent regulator, where the amount of ligand instructs different fates during development (e.g. DPP in the *Drosophila* embryo) and also different growth rates in *C. elegans* (7,10) A potential explanation for the observation that *dbl-1*(gof) does not increase fat accumulation as expected, has been that perhaps the right amount of *dbl-1* expression has to be provided. We favour a model where the right dose of BMP signalling is permissive for *fat-6/7* expression and the expression of fat desaturases in the cold requires additional instructive signals, beyond BMP. We have changed the text (line 459 onwards) and updated the model to reflect these observations (**Figure 6**).

3**.      hsf-1 overexpression in neurons is sufficient and necessary to control fat metabolism in peripheral tissues. However, it is not clear if endogenous hsf-1 also functions in the peripheral non-neuronal tissues to mediate the fat remodelling signalling. Is over-expressing hsf-1 in neurons is still able to regulate fat metabolism in hsf-1(sy441) mutants?**

We attempted the experiment proposed but unfortunately both *fat-7p*:GFP reporters available are on the same chromosome as *hsf-1(sy441)* and so closely linked that we were unable to make the doubles. However, we have performed experiments using a strain that when fed with RNAi downregulates gene expression in neurons (TU3401*: sid-1(pk3321) V; uIs69 [pCFJ190(myo-2p::mCherry*) + unc-119p::sid-1). Using this reagent, we were able to downregulate *hsf-1* in neurons and found that it has a detectable effect on the temperature dependent increase in lipase expression at 25°C (**Figure 5 F-G**) but not of *fat-6/7* expression (**Fig 5 H-I**). In addition -using the same tools- we performed lifespan assays and found that *hsf-1* is necessary in neurons to control lifespan at warmer temperatures (**Figure 5J**). The results have been added to main text line 564 onwards. Future studies should address the point raised by the reviewer: if HSF-1 in other tissues also contribute to *hsf-1* neuroendocrine pathway.

**4.      The authors argue that lipid remodelling caused by hsf-1^neural^ may provide beneficial effect on lifespan at 25ºC. This is a very interesting hypothesis. However, it is not clear if these changes in lipid metabolism are involved in the hsf-1^neural^ induced lifespan extension. It is also possible that hsf-1^neural^ prolongs lifespan through daf-16/FOXO dependent signalling (Douglas et al., 2015), rather than through lipid remodelling. To determine whether lipid remodelling is sufficient to adapt to higher temperatures the authors can test if fat-6/7 and dbl-1 mutations are also able to extend lifespan at 25ºC.**

Our reviewer raises a good point, and to address it we have performed lifespan experiments in *dbl-1*(*nk3*) (NU3) and *fat-6(tm331); fat-7(wa36)* (BX156) mutants. In **Figure S7A**, we report survival curves for these animals grown at 25°C. Loss of the fat desaturases *fat-6* and *fat-7* causes animals to survive longer than control animals at 25C (p<0.0001). **Figure S7B** also shows survival curves for *dbl-1(nk3)* (NU3) animals, where we find that removal of *dbl-1* is beneficial for survival at 25°C, but not as beneficial as *hsf-1^neuro^.* This result is not unexpected as *dbl-1(nk3)* makes worms more susceptible to bacterial pathogenesis (10) Our data suggest that loss of BMP signalling is epistatic to *hsf-1^neuro^* and that removal of this signalling pathway is beneficial for survival at warm temperatures. The results have been reported in lines 568 onwards.

Other points:

1.      **In figure 1f, the authors compare *hsf-1^neural^* and SCD KO phospholipid ratios at 20°C with levels in the wt at 15 and 20C. Adding wild type PE/PC ratio would make this comparison more informative.**

To address our reviewer’s request, we re-measured total amounts of PE and PC in a full series of wild type and *hsf-1^neuro^* worms grown at different temperatures and include these results as the new **Figure 1F.** The graph shows that PE/PC ratio follows an expected trend, increasing in wild type animals as their cultivation temperature increases from 15°C to 20°C to 25°C. The results -although not significant-show a very clear graded temperature-sensitive increment in the ratios with temperature. In the case of *hsf-1^neuro^*, PE/PC ratios are as high as the ratios found in wild type animals grown at 25°C and this ratio is independent of the growth temperature of *hsf-1^neuro^* animals. The key observation is that *hsf-1^neuro^* ratios are significantly different from wild type animals raised at lower temperatures. These results are consistent with our model whereby *hsf-1^neuro^* have the *thermostat* constitutively turned ON independent of the growth temperature and have been added to the main text (line 181 onwards).

**Also, it would be good to determine whether *hsf-neuro* mutants still can modulate the lipid desaturation in response temperature changes or if they are the same at 15, 20 and 25C.**  We agree with the reviewer that this would be an interesting point to make, however, in prioritising the response to reviews, we decided not to pursue this experiment.

**2.      I may have missed it, but I could not find in the methods if the RNAseq experiments were actually done in independent triplicate samples (as is common). This information should be included.** 

The author is right about this point, we have now included the number of replicates (n=3) in the materials and methods line 989.

**3.      In figure 4 the authors attempt to identify neurons responsible for *hsf-1^neural^* fat phenotype by restoring tax-2 function in specific subsets of cells; yet cell specific promoters do not fully rescue. Does tax-2 expressed under the endogenous promotor fully rescue? That seems the first logical experiment to compare it to the partial rescues of more cell specific expression expts. Partial rescue may be caused by mosaic expression.** 

This is a fair point however, we had to prioritise other responses.

Minor points
4.      **I find the use of "nhsf-1 " to refer to expression of a transgene confusing as it closely resembles the nomenclature of gene names. Replacing this with something indicating this is expression of hsf-1 transgene in neurons (i.e. "ExHSF-1n") would make this more obvious.**

We decided to change the acronym to *hsf-1*^neuro^, as it more clearly indicates that this is a transgene and it is more in line with the acronym used in (11).

**5.      In the survival curve (fig 1B) of *hsf-1^neol^* #2 418 animals are censored. Why is this number so much higher than the wild type?**

Similar to *dbl-1(nk3)*; *hsf-1^neuro^* animals tend to bag more than wild type animals. They also leave the plate more often. We have added this technical detail to the methods section (lines 801-2)

**6.      In figure 1d, the "SCD KO" is compared to wild type. Would be more obvious to just give the genotype: fat-6; fat-7 rather than the acronym.**

We have changed this in the figures.

**7.      Figure 3a uses a colour scale with arbitrary min/max values which make the differences seem more striking than they actually are. Would be more appropriate to use a bar diagram with the table values. It would be good to include tax-4 as well in the data.**

We have now used a bar-plot to represent the data. We have used a normalised bar-plot because using average values creates a plot that is very difficult to understand (see figure below and response to reviewer 4). The un-normalised values and statistics are presented in **Table S5**. We have also completed replicates with RNAi against *tax-4*; however, it causes a milder suppression than *tax-2*. We believe this is caused by inefficient RNAi technique because a loss of function allele of *tax-4* acts as a suppressor of *hsf-1^neuro^*, comparable to *tax-2* loss of function alleles.

These two figures try to show why we have used normalised values (second shown figure) instead of average values (first shown figure). However un-normalised values are presented in **Table S6**.

**8.      Sometimes p values are missing from bar diagrams e.g. In figure 3b&c, tax-4 mutants have a higher baseline level of fat-7 expression than wild type. Is this difference significant? Similar Fig 3e wt tax-2, Fig 3f (tax-2 + or - hsf-1neural).**

Thank you for pointing this out. We have tried to simplify figures by only showing p values that are relevant to the ideas presented in the text, while adding a full statistical analysis in the supplementary tables. But in some cases, this can cause confusion.

In **Figure 3C,** the reviewer is right to point out that *fat-7:gfp* is lower in *tax-4(+)* (MOC193) animals than in *tax-4(p678)* (MOC232). As reported in **Table S6**, the mean intensity values are: *tax-4(+)* =11619(+/-867SEM), n=233 versus *tax-4(p678) =*14924(+/-1206SEM), n=224. However, we find that this difference is not statistically significant. We feel that this data is quite robust, as we took 7 biological replicates to make this point. We have added p value to the figure itself and made this point clear in the figure legend.

In **Figure 3E**, and as shown in **Table S8**, in *tax-2(+)* animals the expression of *lipl-3* is 0.007 +/- 0.0022SEM lower than in *tax-2(p671)*, which is 0.02 +/- 0.002SEM. This difference is significant p=0.04 using a one-way ANOVA test. We have added the average values in the figure legends and the p value to the figure.

**9.      In table 2-bodipy assay, there appears to be a typo in the neurons tested column. 'tax-4(+) in AWC…'  should be tax-2.**

We thank the reviewer about noticing this typo, which we have fixed in the Table.

**10.     In figure S2a, the authors show worm area/size and reproduction phenotypes for hsf-1neural, are these suppressed by loss of tax-2/tax-4?**

We were unfortunately not able to complete these experiments, as we tried to address as many points raised by our 4 reviewers as possible in limited time.

**11.     Figure 4h makes the genetic interaction between HSF-1, TAX-2/TAX-4 and DBL-1 quite confusing as the "triple mutant" is statistically identical to both single mutants and to the wild type.**

We appreciate the reviewer’s comments and we have eliminated **Figure 1J** from the main figures and added it to **Sup Fig 6 C**. We think that the triple epistasis is better understood in the context of the two independent pathways that have been found in this study (see text in green). However, given all the new results presented as the result of this revision, we may not need to add this figure at all and would welcome the opinion of the reviewers with regard to this point.

LINE 499 onwards

One potential scenario is that the neurons that activate the HSR at 25℃ are the 12 TAX-2/4 expressing neurons. To determine if this was the case, we performed a co-localisation experiment using two independent reporters (either mORANGE or wrmSCARLET driven by the *tax-4* promoter) in addition to the cGAL system to monitor neurons with an active HSR. As shown in **Figure S6A-B** and in **supplemental movies** the analysis of both reporters indicates no consistent co-localization between TAX-4 neurons and neurons with an active HSR. These observations are consistent with the idea that TAX-2/4 neurons play two distinct roles in the modulation of *hsf-1^neuro^* phenotypes, one directly to control *fat-7* via BMP/SMA and another altered indirectly by TAX-2/4 neurons-to control lipase expression. This idea is also supported by the results obtained by driving *hsf-1* with a *tax-4* promoter (**Figure S2**). To further strengthen this point, we performed a triple epistasis experiment by removing the function of *tax-2* and *dbl-1* from *hsf-1^neuro^* animals. If *tax-2* dependent suppression on fat accumulation was entirely dependent on counteracting *hsf-1^neuro^* -dependent downregulation of TGF-β/BMP signalling, then removing the BMP ligand should prevent *tax-2* suppression entirely. However, we find that *tax-2* can partially rescue the lean phenotype of *hsf-1^neuro^* even in the absence of TGF-β/BMP signalling. The partial rescue is further support for the idea that two independent pathways are influenced by the loss of *tax-2* (**Figure S6C**).

**12.     The authors show induction of hsp16.41 in a subset of neurons in the head, are these neurons that also express TAX-2/TAX-4. This seems to be a central point in their model. A double labelling expt would be easy to do to support their model.** 

Previously addressed.

Reviewer#4:

In this manuscript, the authors described their new findings that neuronal HSF-1 may serve as a thermostat to sense environmental temperature changes and consequently regulate the lipid storage, fat desaturation, and membrane fluidity of peripheral tissues.  Moreover, they have identified a subset of neurons as well as the signalling pathway (i.e. TGF-β/BMP) that may be responsible for this regulation.  This is the first study to demonstrate that membrane composition and fluidity may be cell non-autonomously regulated by HSF-1 activity in a group of sensory neurons.  
Overall, the findings are novel and of great interest to the field.  The mechanistic insights provided here in the manuscript, although still with some loose ends, further enhance the potential impact of this works.  The manuscript will be further improved with some relative minor clarifications and modifications listed below:

We thank our reviewer for their supportive comments.

**1. One important conclusion the authors made is that there are more than six tax-2/tax-4 expressing neurons are responsible for the HSF-1 mediated fat remodelling.  However, the six neurons that tax-2(694) actually affected (AQR and five others) were never examined.  On the other hand, the neurons (ASG, and five others) that are not altered tax-2(694) were actually examined.  Unfortunately, inactivation of individual neuron or a combination of 2 or 3 of this set of neurons does not affect the fat phenotypes.  Since experiments eliminating all six of this set of neurons were not performed, it is hard to come to the conclusion that all six neurons are involved.  It remains possible that eliminating a combination of one or two of the neurons from each set (i.e. the AQR set and the ASG set) is sufficient to completely rescue the phenotype.**

We agree with the reviewer’s point. We only tested in detail 6 of the neurons (single, double and triple) combinations. We tested one of the neurons affected by *tax-2(p694)*, the AFD neurons, but saw no specific rescue. To address this point, we generated new transgenic lines using the *cng-2* and *cng-3* promoters that drive expression in specific subset of 5 or 6 *tax-2/tax-4* neurons, including neurons affected by *tax-2(p694)* partial lof and neurons not affected in *tax-2(p694).* The *cng-2* promoter drives expression in 6 neurons (ASE, affected in *tax-2(p694)* and AWC, ASI, ASJ, ASG and ASK, unaffected in *tax-2(p694*)). The *cng-3* promoter drives expression of *tax-4* in 5 neurons (ASE, AFD affected in *tax-2(p694),* and AWB, AWC, ASI, unaffected in *tax-2(p694).* We generated lines in *tax-4(p678)* background carrying either *cng-2p::tax-4(+)* or *cng-3p::tax-4(+)* or both *cng-2p::tax-4(+)*;*cng-3p::tax-4(+).*

However, and very unfortunately, all lines caused toxicity to the worms and therefore we could not complete this experiment.

**2. In many cases, the authors describe the suppression or rescue of the phenotypes by percentage (both in figure and in the text), which is somewhat confusing.**

We thank the reviewer for pointing this out, whilst the majority of the figures present average values (with some exceptions that we have now changed), the main text does refer to changes perceptually. We are not entirely sure how to make the comparisons clearer, but we have changed the phrasing of problematic sentences and also added -wherever possible- the average values and standard deviations of key data within the figure legend.

Changes have been highlighted in red in the legends.

In Figure 2 legend: “Mean intensity fluorescence levels are reduced by 29.3% and 40% in *hsf-1^neural^#1* (mean intensity of 4731(+/-479SEM) versus 3343 (+/- 340 SEM) and *# 2*, (mean intensity of 4231(+/- 79 SEM) versus 2554 (+/- 268 SEM) respectively, indicating that *hsf-1^neural^* animals are leaner than WT controls”.

**For example, in line 303 the authors state that " the loss of tax-2 function causes an 80% suppression".  This is similar to what is presented in Figure 3A.  However, in the figure 3 legend, the same result was described as follows "for the control…., the difference between sibling is 37%, this difference is only 10%......”.]**

We thank the reviewer for pointing this out. In general, we avoid using percentages throughout the figures, and almost all data has been presented as average values. However, it is true that **Figure 3A** was represented as a heat map, and that representation can be misleading, so we have replaced that figure by a bar plot using values normalised to controls (shown in the response to Reviewer 3). We initially sought to avoid using this sort of normalisation of the data, however if we present unnormalized data, we fear that readers will not get the important point that we are trying to make. The un-normalised values are available in **Table S5**.

The changes added to the text:

Line 289

Among them, the loss of *tax-*2 function acts as a strong suppressor of the effect that Ex hsf-1 has on *fat-7* expression (**Fig 3A**).

Figure 3, line 299

The graph compares mean expression values of RNAi-fed *Ex hsf-1^neural^*(+/-) pairs, where the average mean intensity values of GFP driven by *fat-7p::GFP* in animals containing the *Ex hsf-1^neural^* array were normalised to controls lacking the array. While most treatments do not change the effect of *Exhsf-1^neural^ -*which causes a 37% drop in *fat-7p:GFP* fluorescence- the treatment with *tax-2* RNAi makes animals almost entirely refractive to the presence of *Exhsf-1^neural^* and the 19% difference among the *Exhsf-1^neural^* pairs (+/- transgene) is not significant.

Even in the presence of *Exhsf-1^neural^*, GFP expression in *tax-2* treated animals are not significantly different with regards to controls.

**Similarly, this is also the case in Figure 4H-I, and line 503-505.  I find it hard to comprehend.  It may be a good idea to present the data in a more straightforward way.**

See reply below.

**3. Also in line 470, the authors claimed that "In the absence of dbl-1 function, tax-2(p671) restores 30% less fat accumulation than in the presence of dbl-1 function."  I am not sure how this is calculated, since I do not see data for hsf-1neural #2; dbl-1(nk3) double mutants.  Moreover, the difference between hsf-1neural; dbl-1; tax-2 triple mutants and hsf-1neural singles seems to be not statistically significant (labelled as "ns" in Figure 4H).** 

We appreciate the reviewer’s comments and we have eliminated **Figure 1J**. These results make more sense now that we have done additional experiments in response to the review process. It is now presented in Line 500 onwards, as part of **Figure S6**.

**4. Is the experiments for Fig 1D and 1E done at 15°C or 25°C?  For Figure 1F, a WT control at 20°C may be needed as a proper control for the mutants.**

To respond to the reviewer’s request, we have re-measured total amounts of PE and PC in a full series of wild type and *hsf-1^neuro^* worms grown at different temperatures and include these results as the new **Figure 1F.** The graph shows that PE/PC ratio follows an expected trend to increase in wild type animals at 15°C, 20°C and 25°C. The results show a very nice graded increment with temperature. In the case of *hsf-1^neuro^*, PE/PC ratios are as high as the ratios found in wild type animals grown at 25°C and the ratios are independent of the growth temperature of *hsf-1^neuro^* animals. The key observation is that ratios in *hsf-1^neuro^* are significantly different from ratios of wild type animals raised at lower temperatures. These results are consistent with our model whereby *hsf-1^neuro^* have the thermostat constitutively turned ON independent of the growth temperature. The Figure legend and the main text from line 181 onwards have been modified.

**5. Line 400-402.  The authors state that they have tested both ttx-1 and ttx-3 in Fig 3H and Table 2.  But I cannot find any data for ttx-3**.

We have added the *ttx-1* and *ttx-3* data to **Table S6**.

**References**

1. Calixto A, Chelur D, Topalidou I, Chen X, Chalfie M. Enhanced neuronal RNAi in C. elegans using SID-1. Nat Methods. 2010

# 2. Omi S, Pujol N *unc-119* mutants have an increased fungal spore adhesion that is not rescued by *Cb-unc-119*. Micropublication Biology, 2021 DOI: [10.17912/micropub.biology.000344](https://doi.org/10.17912/micropub.biology.000344)

3. Stefanakis. N, Carrera I, Hobert O. Regulatory logic of pan-neuronal gene expression in *C.elegans*. Neuron 2015; 87:733-750. http://dx.doi.org/10.1016/j.neuron.2015.07.031

4. Venkatachalam V, Ji N, Wang X, Clark C, Mitchell J, Klein M, Tabone C, Florman J, Ji H, Greenwood J, Chisholm A, Srinivasan J, Alkema M, Zhen M, Samuel A. Pan-neuronal imaging in roaming *C.elegans*. PNAS 2016;113(8): E1082-E1088

<https://doi.org/10.1073/pnas.1507109113>

5. Hutter H, Suh J. GExplore 1.4: An expanded web interface for queries on Caenorhabditis elegans protein and gene function . Worm [Internet]. 2016;5(4):e1234659. Available from: http://dx.doi.org/10.1080/21624054.2016.1234659

6. Ebbing A, Vértesy Á, Betist MC, Spanjaard B, Junker JP, Berezikov E, et al. Spatial Transcriptomics of C. elegans Males and Hermaphrodites Identifies Sex-Specific Differences in Gene Expression Patterns. Dev Cell. 2018;47(6):801-813.e6.

7. Suzuki Y, Yandell MD, Roy PJ, Krishna S, Savage-Dunn C, Ross RM, et al. A BMP homolog acts as a dose-dependent regulator of body size and male tail patterning in Caenorhabditis elegans. Development. 1999;126(2):241–50.

8. Zhang X, Zhang Y. DBL-1, a TGF-β, is essential for Caenorhabditis elegans aversive olfactory learning. Proc Natl Acad Sci U S A. 2012;109(42):17081–6.

9. Clark JF, Meade M, Ranepura G, Hall DH, Savage-Dunn C. Caenorhabditis elegans DBL-1/BMP regulates lipid accumulation via interaction with insulin signaling. G3 Genes, Genomes, Genet. 2018 Jan 1;8(1):343–51.

10. Gumienny TL, Savage-Dunn C. TGF-β signaling in C. elegans. WormBook. 2013;1–34.

11. Douglas PM, Baird NA, Simic MS, Uhlein S, McCormick MA, Wolff SC, et al. Heterotypic Signals from Neural HSF-1 Separate Thermotolerance from Longevity. Cell Rep. 2015 Aug 18;12(7):1196–204.
