# Supplementary material for: Neuronal HSF-1 coordinates the propagation of fat desaturation across tissues to enable adaptation to high temperatures in C. elegans
Source: PLoS Biol. 2021 Nov 1;19(11):e3001431. doi: 10.1371/journal.pbio.3001431 (PMC8585009; doi:10.1371/journal.pbio.3001431)
Supplement: Attachment — Submitted filename: Final Response to Reviewers.docx [file pbio.3001431.s024.docx]

**Request from editors:**

1) DATA REQUEST: Please provide the underlying data for each figure in your manuscript, including supplemental figures. This can be provided as a supplemental file or by uploading the data to a publicly available repository. **Please make sure to reference the underlying data in each figure legend. For example you can add the sentence "The data underlying this figure can be found in S1_Data". Please find more detail on this request and on our data policy below my signature.

We provide all underlying data per figure and have uploaded it to the following open-access repository:

https://zenodo.org/record/5524609#.YUx0eS1Q3tE

2) DATA REQUEST: Thank you for uploading the RNA-seq data files from WT and neuro-shf1 to NCBI GEO. Can you please provide me with a reviewer token so that I can access the data and confirm that it complies with our data availability policy? (Apologies if you have already included the token and I somehow missed it).

The data accession is GSE145123 and can be viewed in private mode here:

**https://www.ncbi.nlm.nih.gov/geo/query/acc.cgi?acc=GSE145123
Token:** abitckaqdbyzbed

The data will be released as soon as the journal provides a publication date.

**Reviewer remarks:**

**Reviewer #1:** The authors have revised the manuscript extensively and addressed many of the questions from reviewers. However, the key concern on the role of endogenous hsf-1 in neurons, also raised by another reviewer, still remains. The authors used the strain TU3401:sid-1(pk3321)V,uIs69 [pCFJ190(myo-2p::mCherry) + unc-119p::sid-1), which is neuronal RNAi sensitized yet without evidently blocking RNAi from other tissues (in particular, the intestine). Thus it is still possible that endogenous neuronal Hsf plays negligible role. Without more definitive evidence from neuronal specific loss-of-function and rescue of hsf-1, the findings may be limited by the overexpression effect of hsf-1 and interpretation of such limitation and caveats should be at least discussed explicitly in the paper.

We thank the reviewer for raising this important point and we have changed the text as follows from line 566 onwards:

“If the gene expression changes caused by neuronal *hsf-1* KD were functional, these should impair the survival of worms at warmer temperatures. We observe that this is the case because *hsf-1* KD in a strain where RNAi is effective primarily in neuronal cells, shortens lifespan at 25℃ compared to controls. The KD of *hsf-1* only changes lipase levels and does not change *fat-6/7* expression levels, so the reduced advantage at 25℃ must be caused by decreased lipase levels in the gut. However, mutants lacking *fat-6* and *fat-7* (**Fig S7A**); or *dbl-1* function (**Figure S7 B**) also shorten lifespan at 25℃, suggesting that both enzymatic branches controlled by *hsf-1^neuro^* are key to survival at higher temperatures. However, this conclusion has to be drawn cautiously because although we controlled for known spurious effects of the RNAi strain in the hypodermis (**Figure S8**), unreported leakage to other tissues may have an influence on the loss of function phenotypes observed.”

**Reviewer #2**: As I stated in the first review of the manuscript, the findings presented here are solid and of novelty and importance even if the mechanistic understanding is still somewhat patchy. The authors have gone to great length to address the concerns of all reviewers and in light of new data they have refined their model and interpretations, so the manuscript has certainly improved. I have no more concerns.

We thank reviewer for her/his supportive take on the article and the revision.

**Reviewer #3**: The authors have performed additional experiments that improved the manuscript. The role of hsf-1 and TAX-2/4 neurons as a thermostat at physiological conditions were clarified.


- Although some minor points were not completely addressed due to time constraints, I think that the key conclusions of this study are now well supported in the revised manuscript

We thank reviewer for her/his supportive take on the article and the revision.

Some remaining issues

The normalized bar diagram as shown in the  the rebuttal should replace fig 3a . Also, the the tax-4 data should be included in fig 3A. Maybe this is an oversight since the authors claim in the rebuttal that 3a was replaced?

We apologise, this was indeed an oversight and have now changed the figure accordingly.

Figure 4H and 6 seem a duplicitous.

We agree with the reviewer and have decided to remove the diagram in figure 4.

**Reviewer #4**: In this revised manuscript, the authors have made a lot of efforts to address the concerns that all four reviewers raised.  Most importantly, the model has been modified based on newly obtained data.  For example, the roles of TAX-2/TAX-4 neurons in regulating fat remodeling have been further clarified.  Unfortunately, due to technical limitations and time constrain, there are several questions remain unanswered.  Overall, I think the manuscript in its current form, even with those unanswered questions, still provided sufficient novel findings that warrant its publication.

We thank reviewer for her/his supportive take on the article and the revision.
